# Deep learning-based estimation of Flory–Huggins parameter of A–B block copolymers from cross-sectional images of phase-separated structures

**DOI:** 10.1038/s41598-021-91761-8

**Published:** 2021-06-10

**Authors:** Katsumi Hagita, Takeshi Aoyagi, Yuto Abe, Shinya Genda, Takashi Honda

**Affiliations:** 1 Department of Applied Physics, National Defense Academy, 1-10-20 Hashirimizu, Yokosuka, 239-8686 Japan; 2 Research Center for Computational Design of Advanced Functional Materials, National Institute of Advanced Industrial Science and Technology, Central 2, 1-1-1, Umezono, Tsukuba, Ibaraki 305-8568 Japan; 3 Zeon Corporation, 1-2-1 Yako, Kawasaki-ku, Kawasaki, 210-9507 Japan

**Keywords:** Information technology, Chemical physics, Molecular self-assembly, Self-assembly, Self-assembly

## Abstract

In this study, deep learning (DL)-based estimation of the Flory–Huggins χ parameter of A-B diblock copolymers from two-dimensional cross-sectional images of three-dimensional (3D) phase-separated structures were investigated. 3D structures with random networks of phase-separated domains were generated from real-space self-consistent field simulations in the 25–40 χ*N* range for chain lengths (*N*) of 20 and 40. To confirm that the prepared data can be discriminated using DL, image classification was performed using the VGG-16 network. We comprehensively investigated the performances of the learned networks in the regression problem. The generalization ability was evaluated from independent images with the unlearned χ*N*. We found that, except for large χ*N* values, the standard deviation values were approximately 0.1 and 0.5 for A-component fractions of 0.2 and 0.35, respectively. The images for larger χ*N* values were more difficult to distinguish. In addition, the learning performances for the 4-class problem were comparable to those for the 8-class problem, except when the χ*N* values were large. This information is useful for the analysis of real experimental image data, where the variation of samples is limited.

## Introduction

Artificial intelligence (AI) and deep learning (DL) algorithms are expected to improve scientific research^1–6^. For example, their application for COVID-19 diagnosis has received considerable interest^7–9^. In addition, AI and DL are expected to serve as quantitative measurement methods for images obtained in experiments in research works related to polymer materials. Although material discovery based on physical properties using machine learning (ML) has been investigated in many studies^10–19^, relatively limited research has been conducted on DL for images^20^. Up until now, image classification and super-resolution processing have been the major tasks in DL for experimental images. In material science, the application of DL for image classification^21–27^ and super-resolution processing^28–33^ has been extensively reported. Recently, many simulation-based studies on the inverse design via a generative adversarial network (GAN) with forward analyses of DL have been reported^34^. Hiraide et al.^34^ tried DL-based design of phase-separated structures as continuums in two-dimensional (2D) space; however, for polymer materials, three-dimensional (3D) nanostructures are more desirable. In addition to the relationship between 3D nanostructures and mechanical properties, the effects of atomic- and molecular-level compositions and material processes in the formation of 3D nanostructures must be elucidated. For polymer materials, 2D images of stained specimens can be easily obtained using electron microscopes; however, 3D images can be obtained only via costly, time-consuming methods such as tomography^35–37^. Thus, for research aimed at the development of polymer materials, developing a technology to establish a connection between experimental images and simulations of polymer materials with high accuracy is considered very important.

The superior DL-based image-classification performance seen at the Large-Scale Visual Recognition Challenge, 2012^38^, has paved the way for the current AI trend: AlexNet^38^ achieved improvements over traditional convolutional neural networks (CNNs), and it consists of five convolutional layer blocks and three fully connected layers. Since then, superior algorithms such as VGG-16 and VGG-19^39^, ResNet^40^, GoogLeNet/Inception^41^, Xception^42^, MobileNet^43^, and DenseNet^44^ have been proposed. As a general example, these networks have been used to estimate the age of a person from a photograph of their face.

According to textbooks and leading papers^45–54^, the phase diagram of a block copolymer (BCP) melt is determined by two independent parameters: χ*N* (where χ is the Flory–Huggins interaction parameter and *N* the total number of segments in a BCP chain) and the A-component fraction *f*. For the A–B diblock copolymer, $N={N}_{\mathrm{A}}+{N}_{\mathrm{B}}$ and $f={N}_{\mathrm{A}}/N$. Generally, average structures with high symmetries, such as gyroid, cylinder, lamellar, and sphere phases, have been confirmed via small-angle scattering experiments^54^. Although highly controlled experiments have achieved high regularities, the actual materials may still have defects and mesoscale structural distortions. For these highly symmetric structures, such as lamellar structures, domain spacing is linked with the effective *χ* parameter^55–66^. Here, the effective *χ* parameter is controlled using chemical composition and chain architecture. The *χ* value is considered to affect the process of phase separation and characterize the morphology of the interface. Therefore, it is expected that *χ* can be estimated from the morphology information during the phase separation process.

Advanced controls in the phase-separated structure of BCPs are important for industrial applications, such as directed self-assemblies (DSAs) for semiconductor processes and soft materials such as high-performance mechanical rubbers. Concerning DSA^67–70^, regular lamellar and cylindrical structures were required for line/space arrangements and contact-hole patterning, respectively. The implementation of DSAs on substrates has involved a rigorous study of nanoimprinting lithography^71^, electron beam lithography^72,73^, and solvent-vapor annealing^74–76^. Solvent-vapor annealing requires effective interactions among polymers, substrates, and solvents^74–76^. To achieve sub-20 nm periods, the control of high χ parameters with custom chemical synthesis is important^77,78^. In contrast, to obtain the optimum mechanical response to the deformation of high-performing soft materials, skillful controls of the “*random network of phase-separated domains*” frozen in a non-equilibrium state are needed^79,80^. Here, asymmetric styrene-isoprene-styrene tri-block copolymers were used to obtain industrial materials with high elasticity and moduli. Morphologies of asymmetric tri-block copolymers have been extensively investigated^81–87^. Recently, Aoyagi^27^ investigated the DL-based predictions of phase diagrams^82^. Note that the relationships between morphologies and mechanical properties under stretching were investigated using coarse-grained molecular dynamics simulations^88–90^. Further research on the random network of phase-separated domains governed by χ*N* is of great interest for the development of high-performing soft materials; estimating χ*N* values from the observed images is an impactful way to enhance this research. Since analytical approaches were limited for non-equilibrium states compared to a well phase-separated structures, χ*N* estimation using AI techniques such as ML and DL is recommended.

The estimation of these two characteristic parameters (χ*N* and *f*) from cross-sectional images of 3D structures is desirable for analyzing experimental images. Discrimination of images with different *f* values is a relatively easy problem when image sizes are not small because this problem corresponds to the estimation of volume density from surface density of images. However, it is not clear whether images with the same *f* value but different χ*N* values can be discriminated. The problem of estimating χ*N* from images is a simple and fundamental problem in the experimental science of materials. The interaction parameter, χ is important for understanding the solubility and microphase separation structures of various polymer chains. The value of χ*N* can be experimentally determined from the correspondence with theoretical prediction by obtaining a highly symmetric structure in a highly controlled experiment and creating a precise phase diagram^54^. However, it would be very efficient if χ*N* could be determined from images of non-equilibrium phase-separated structures, which are easy to observe. In this study, we investigated the basic relationship between the accuracy and errors of χ*N* estimation.

On one hand, if the relationship between a certain feature of cross-sectional images and estimated χ*N* is simple (e.g., linear relationship), the interpolation-estimation accuracy is higher for a lower mean absolute error (MAE) in regression training. On the other hand, if the relationship is not simple, a significantly low MAE through regression training leads to overfitting, wherein the error in the estimation of χ*N* becomes large. Herein, we clarify the type of relationship between a certain feature of cross-sectional images and estimated χ*N*.

χ*N* can be estimated from local high-resolution images by observing the concentration gradient at the interface and/or the interfacial width. Theoretically, the interfacial width is expected to be of the form (χ*N*)^−0.5^ in the weakly segregated region^91^. However, there exists a problem: the density-gradient information is lost owing to staining, which is indispensable for electron-microscope observation, and the binarized image only contains morphology information. This binarization problem is considered more serious than familiar image problems such as those related to noise and focus. In this study, we examined the potential of estimating χ*N* from the morphology information and density profiles in cross-sectional images of global 3D nanostructures with interfacial width of a small number of pixels. Regarding binarized images of stained specimens observed via transmission electron microscopy (TEM), an AI technique that performs estimations only from morphology without a density profile is developed.

## Results

### Data characterization through Image classification

**Figure 1:**
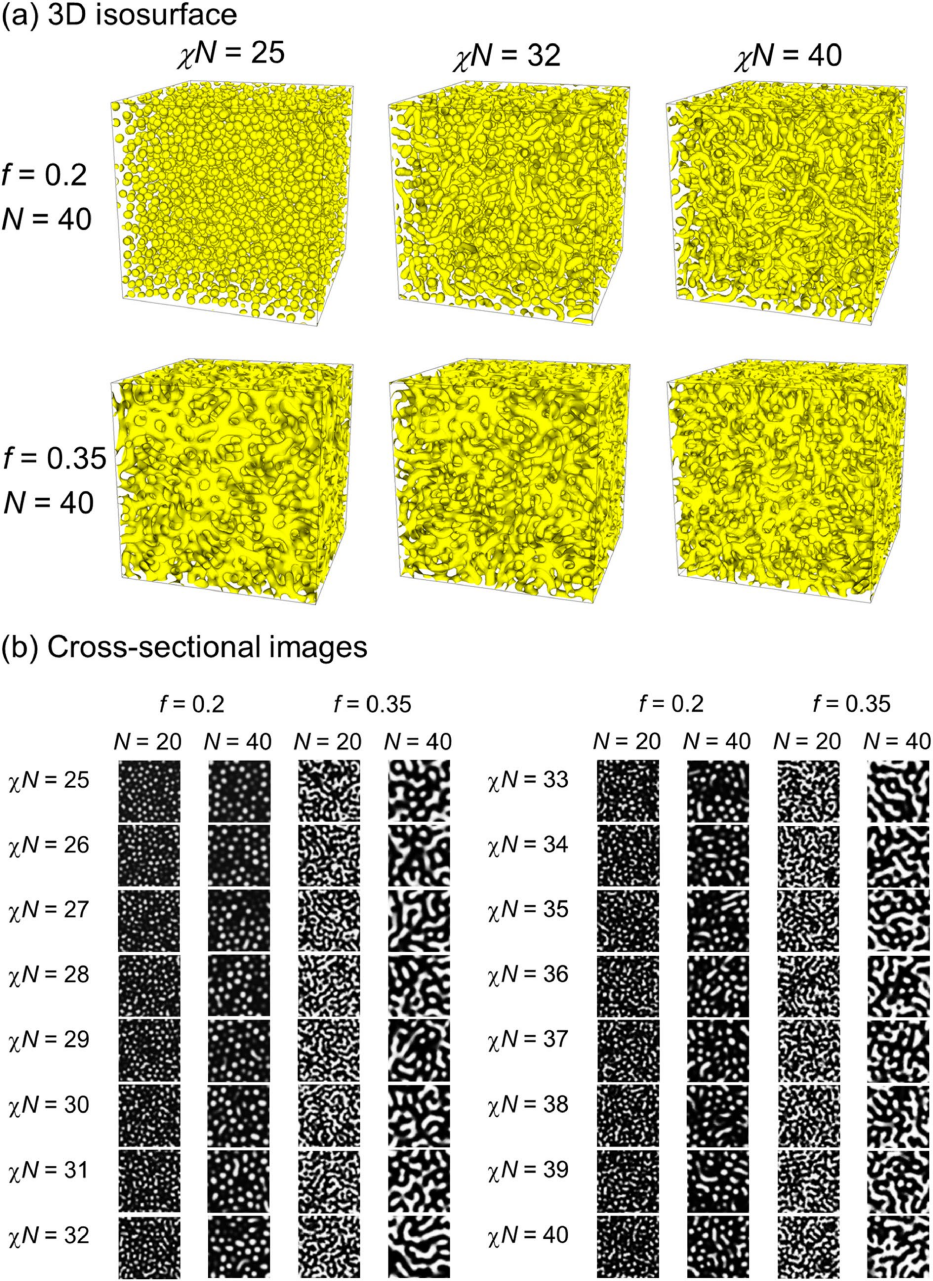
Snapshots of generated structures. (**a**) 3D isosurface and (**b**) cross-sectional images.

To generate image data with $f=0.2$ and $0.35$, 3D field data of the phase-separated structure of A-B BCP were obtained using OCTA/SUSHI^92,93^ based on the real-space self-consistent field (SCF) calculation^52,53^. Figure 1 shows examples of the images generated for $\chi N\ge 25$. A lower value of $\chi N$ was chosen based on the mean field prediction^94^ for $f=0.2$. We obtained density fields after convergence of the SCF calculation or 100,000 SCF steps. Although the highly symmetric structure of triply periodic minimal surface (TPMS) was reported by the experiments, SCF calculations were performed in this study to obtain “random network of phase-separated domains*.*” Although cell-size optimization^95^ is required to avoid the system-size effect under periodic boundary conditions (PBCs) in case of structures with high symmetries, this study did not optimize for the same and instead used a PBC box of fixed size. Conceptually, it is considered that a highly symmetric structure can be obtained by using a sufficiently large system size or by optimizing the system size. From another point of view, the images shown in Fig. 1 can be regarded as structures trapped in the metastable state during the phase separation process. Although these images are not trivial and have certain complexities, they have features that are governed by the interaction parameter, χ*N*. Although the highly symmetric structure under TPMS can be classified by a mathematical index such as the Betti number, there is no mathematical index to classify and express these metastable features. This absence may reveal a case where ML is difficult but DL may prove to be successful. As a result, these images were considered to be suitable to evaluate the potential of estimating χ*N* from their morphology information and density profiles.

For understanding the basic characteristics of the examined data system, image classification was performed before regression. We performed the image classification using Keras^96^ and TensorFlow^97^ packages based on the VGG-16 network. For performance comparison, we performed several ML-based image classifications using Scikit-learn^98^. In the ML-based image classifications, we used support vector machine (SVM) with a radial basis function (rbf) kernel for two features: (1) the histogram of brightness and (2) the histogram of oriented gradients (HoG). In the DL-based image classifications, binarized images were also examined for comparison. To summarize, the present work performed the following image classifications:

1. ML with SVM for histogram of brightness
2. ML with SVM for HoG features
3. DL with VGG-16 for binarized images
4. DL with VGG-16

First, to confirm the superior performance of the VGG-16 model for image classification, we estimated learning curves until 100 epochs and confusion matrices at 100 epochs. For $f=0.2$ and $0.35$, and $N=20$ and $40$, three problems were investigated: (1) 4-class problem with $\chi N=25, 30, 35,$ and $40$; (2) 6-class problem with $\chi N=25, 28, 31, 34, 37,$ and $40$; and (3) 8-class problem with $\chi N=26, 28, 30, 32, 34, 36, 38,$ and $40$. To avoid redundancy, results for the 6- and 8-class problems are presented in Section S1 of the Supplementary Information.

**Figure 2:**
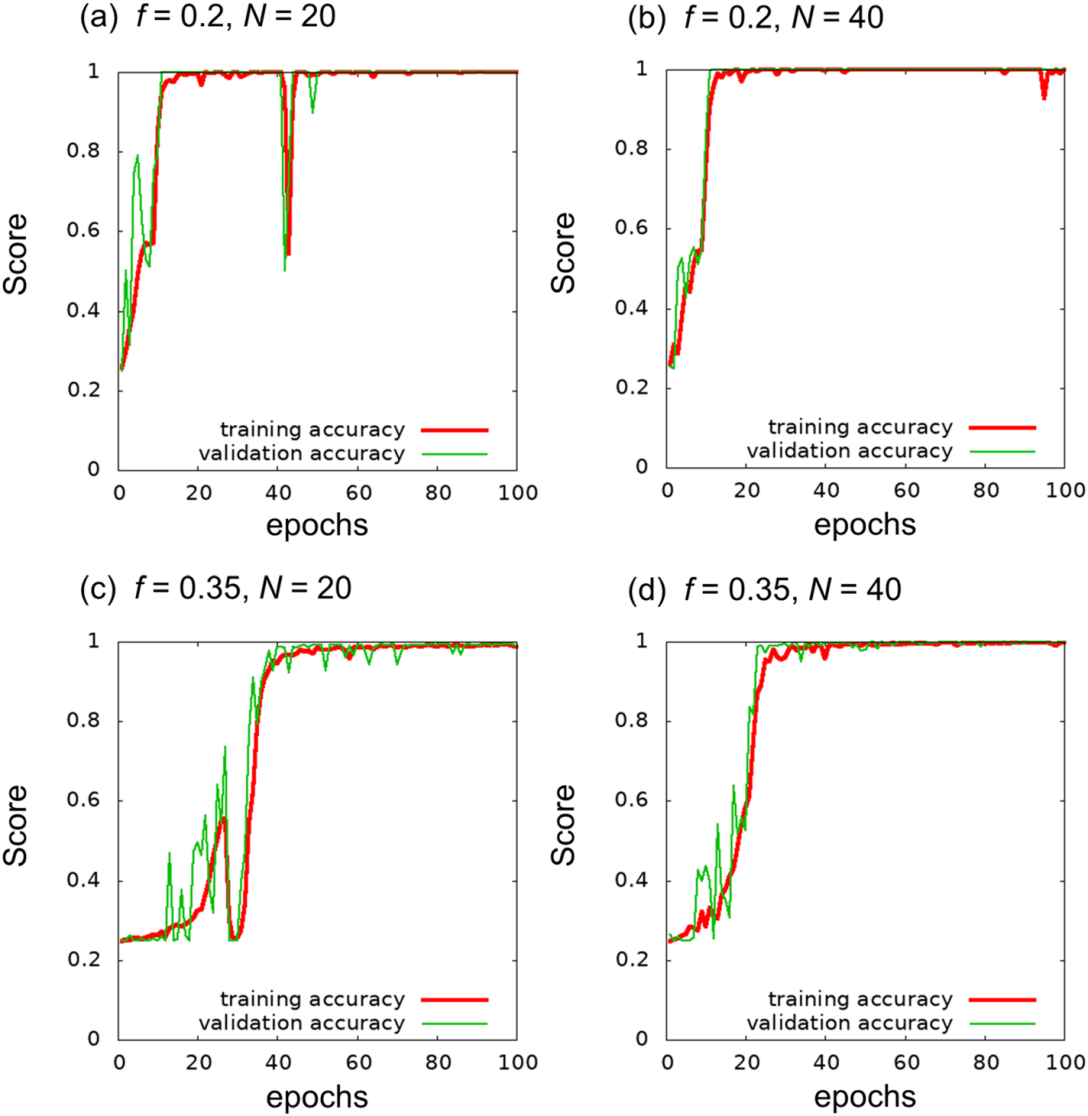
Learning curves of the 4-class image classification under training until 100 epochs.

Figure 2 shows the learning curves of the trainings performed. We found that 100 epochs are enough to obtain a reasonable accuracy. Comparisons among *f* and *N* suggest that training for $f=0.2$ is less difficult than training for $f=0.35$. For $f=0.35$, training with $N=20$ appears to be more difficult than that with $N=40$.

Table 1 presents confusion matrices of the 4-class problem at 100 epochs. For confusion matrix ${M}_{i,j}$, accuracy $A=\sum_{i}{M}_{i,i}/\sum_{i,j}{M}_{i,j}$ and error rate $E =1-A$. For $f=0.2$, $E=1.25 \times {10}^{-4}$ and $0.0$ for $N=20$ and $40$, respectively. When $f=0.35$, $E=1.13 \times {10}^{-2}$ and $1.63 \times {10}^{-3}$ for $N=20$ and $40$, respectively. It was found that *E* for $f=0.2$ is lower than that with $f=0.35$. This tendency is also found in the 6- and 8-class problems presented in Section S1 of the Supplementary Information. These behaviors suggest that the images for $f=0.35$ are more difficult to learn than for $f=0.2$.

**Table 1:** Confusion matrices of the 4-class problem at 100 epochs.

(a) *f* = 0.2, *N* = 20		Estimated χ*N* class			
		25	30	35	40
Actual	χ*N* = 25	2000	0	0	0
	χ*N* = 30	0	2000	0	0
	χ*N* = 35	0	0	1999	1
	χ*N* = 40	0	0	0	2000

(b) *f* = 0.2, *N* = 40		Estimated χ*N* class			
		25	30	35	40
Actual	χ*N* = 25	2000	0	0	0
	χ*N* = 30	0	2000	0	0
	χ*N* = 35	0	0	2000	0
	χ*N* = 40	0	0	0	2000

(c) *f* = 0.35, *N* = 20		Estimated χ*N* class			
		25	30	35	40
Actual	χ*N* = 25	1985	15	0	0
	χ*N* = 30	0	1967	33	0
	χ*N* = 35	1	2	1966	31
	χ*N* = 40	0	0	8	1992

(d) *f* = 0.35, *N* = 40		Estimated χ*N* class			
		25	30	35	40
Actual	χ*N* = 25	1999	1	0	0
	χ*N* = 30	2	1997	1	0
	χ*N* = 35	0	2	1992	6
	χ*N* = 40	0	0	1	1999

The results of the 8-class problem, presented in Section S1of Supplementary Information, suggest that the accuracy for a larger χ*N* is lower when $f=0.2$. This tendency is maintained for $(f,N)=(0.35, 20)$, although it is not clear for $(f,N)=(0.35, 40)$ because of the large error. This tendency is consistent with that of the 4-class problem in Table 1. We expect that the accuracy of each class group on χ*N* in the image-classification problems corresponds to the error of the estimated χ*N* values in the regression problems.

Next, for comparison, we performed ML-based image classifications. Table 2 presents the error rates of the 4-class problem with SVM for the histogram of brightness and the HoG features. Here, 6000 and 2000 images for each χ*N* class were used for the training and evaluation of generalization ability, respectively. Moreover, DL-based image-classification results for binarized images are presented for later consideration. The results for the 8-class problem are also presented in Table 3. The confusion matrices for the 4- and 8-class problems are presented in Sections S2 and S3 of the Supplementary Information. The DL-based image classification exhibits highly superior performance (low error rate) compared to that achieved with ML. Therefore, we consider that regression by ML is not realistic for these datasets. Moreover, it is clear that DL for binarized images outperforms ML.

**Table 2:** Error rates for image classification for the 4-class problem.

	*f* = 0.2		*f* = 0.35	
	*N* = 20	*N* = 40	*N* = 20	*N* = 40
(1) ML-based for histogram of brightness	0.525	0.512	0.150	0.254
(2) ML-based for HoG features	0.512	0.748	0.545	0.612
(3) DL-based for binarized images	0.134	0.137	0.168	0.251
(4) DL-based	1.25 × 10^–4^	0.0	1.13 × 10^–2^	1.63 × 10^–3^

**Table 3:** Error rates for image classification for the 8-class problem.

	*f* = 0.2		*f* = 0.35	
	*N* = 20	*N* = 40	*N* = 20	*N* = 40
(1) ML-based for histogram of brightness	0.763	0.757	0.299	0.387
(2) ML-based for HoG features	0.719	0.721	0.791	0.805
(3) DL-based for binarized images	0.455	0.442	0.515	0.515
(4) DL-based	2.50 × 10^–4^	4.94 × 10^–4^	1.46 × 10^–2^	1.09 × 10^–2^

ML results for the brightness histogram suggest that the prepared images for $f=0.35$ are more dependent on brightness than the images for $f=0.2$. The error rate of ML for the HoG feature for these images is worse than that for the histogram of the brightness. These ML models exhibit inferior performance because the area of each image is small. The image-classification performance improves for larger image sizes in both ML and DL models. One of the authors^99^ investigated the effect of image size on generalization ability of image classification for morphologies of nanoparticles in rubber matrices, where the morphologies were modeled based on the ultra-small X-ray scattering spectrum^100^.

These image-classification results confirm that the prepared dataset has some features that can be distinguished by DL; however, the performance of ML was not good. In the next section, we have used these datasets for the regression problem in the estimation of the Flory–Huggins χ parameter.

### Regression to estimate the Flory–Huggins parameter

As mentioned previously, to investigate the characteristics of regression to estimate the Flory–Huggins χ parameter, we performed regression using the VGG-16 model. When preparing training images via electron microscopy for actual materials, such as stained phased-separated diblock copolymers, the number of prepared materials for the observations is limited to a small value (e.g., less than a few tens of specimens). Thus, in turn, the number of χ*N* classes is limited to a small value. Therefore, in the present regression problem, discrete χ*N* rather than continuous χ*N* is used for the training images. Here, we considered the 8-class problem with training images of $\chi N=26, 28, 30, 32, 34, 36, 38,$ and $40$. In the classification problem, the generalization ability was evaluated from independent images that belonged to the same χ*N* classes and were independent of the training images. For the regression problem, two types of generalization abilities can be evaluated from (1) independent images generated with the same χ*N* value (the 8-classes) and (2) independent images with unlearned χ*N* value. Here, we selected $\chi N=27, 29, 31, 33, 35, 37,$ and $39$ as the unlearned χ*N* values. In this study, we evaluated these two generalization abilities.

**Figure 3:**
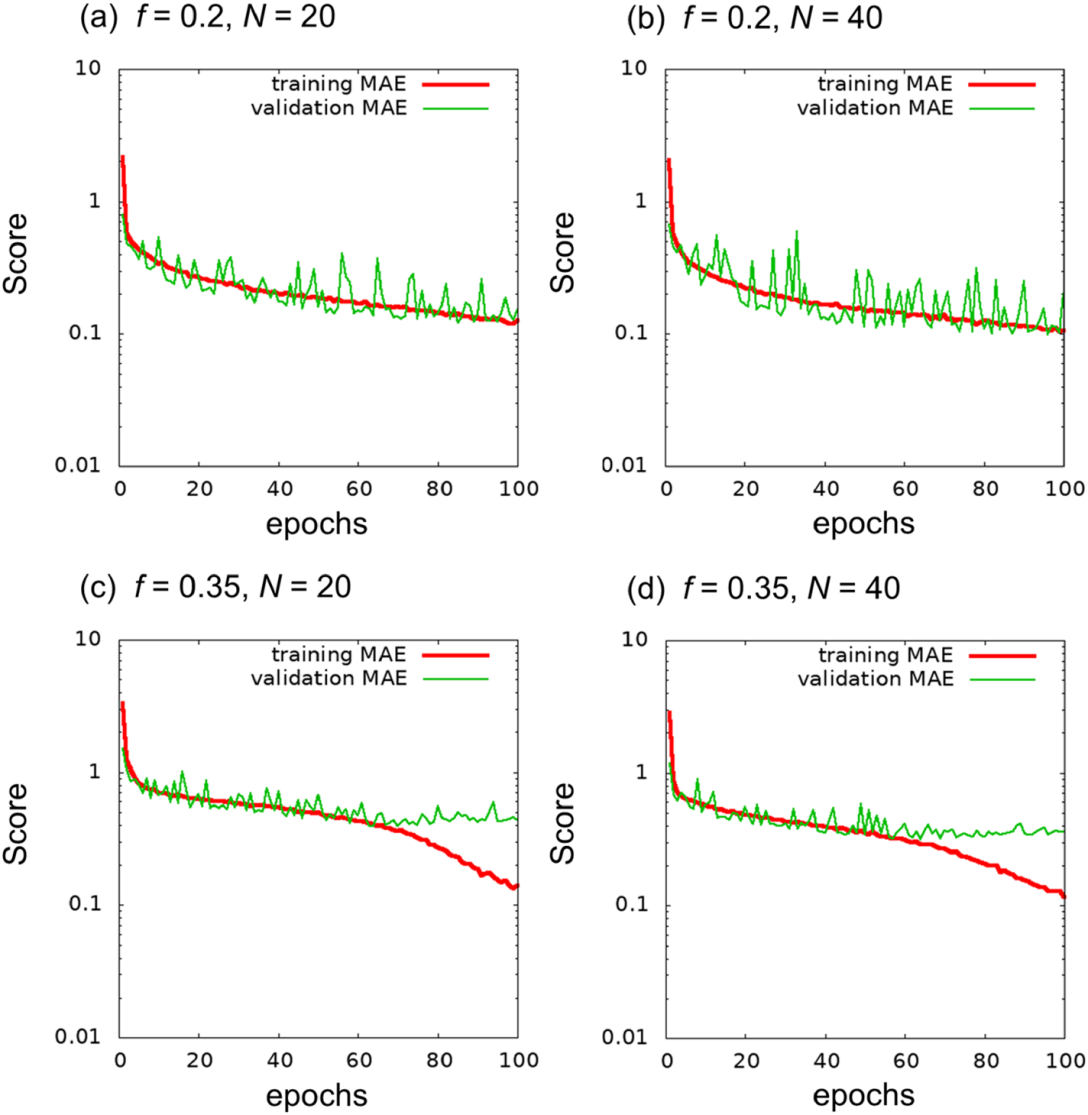
Learning curves of the regression problem until 100 epochs.

As a first test, we performed training with 100 epochs. Figure 3 presents the learning curves until 100 epochs. At $f=0.35$, a discrepancy between training MAE and validation MAE was observed, although a similar discrepancy was not observed for $f=0.2$. We consider that learning from the given training images was saturated (i.e., overfitting tendency). In the learning curve of the validation MAE, the trend comprising the minimum and a subsequent increment can be considered as an indicator of overfitting. In the cases of Fig. 3c and d, the curve around 60 epochs appears to be the minimum. For comparison with the learned network before overfitting, we present the results of an independent run with 50 epochs in Section S4 of the Supplementary Information.

**Figure 4:**
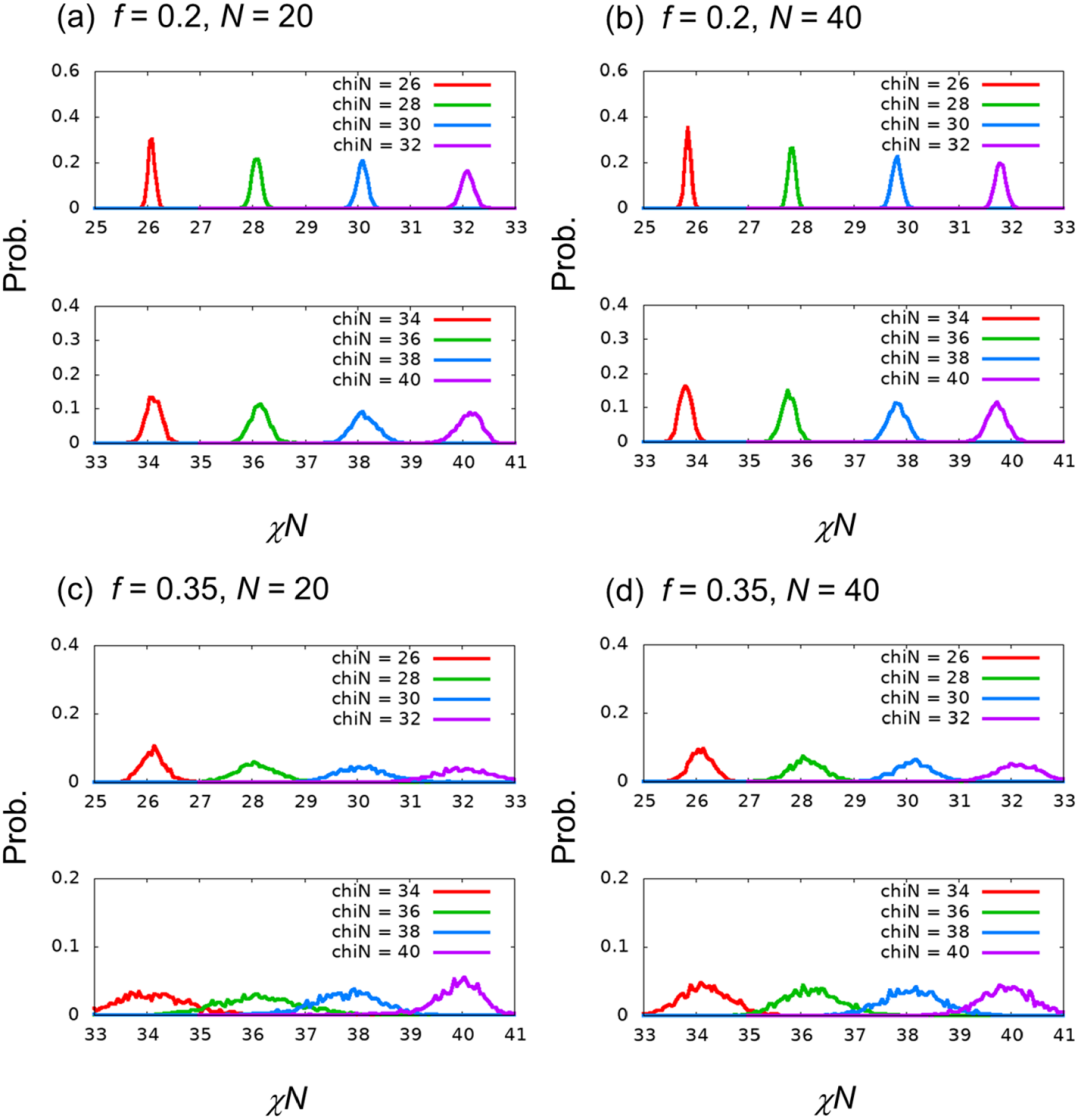
Probability distribution functions of the estimated χ*N* value for evaluation data generated with same χ*N* values as the teaching data. Here, the size of each bin was 0.05.

Figure 4 presents distributions of the estimated χ*N* for independent images whose χ*N* values are the same values as those for the training images. The distribution proceeds differently at $f=0.2$ and $0.35$. These tendencies are the same as those in the image-classification problem as mentioned in the previous section, and they are considered to be related to the difficulty encountered in estimating the χ*N* value. The behavior is similar to that of an independent run with 50 epochs presented in Section S4 of the Supplementary Information.

Table 4 presents the average and standard deviation values of the estimated χ*N* for each χ*N* class. In all cases, the absolute value of the difference from the true value is approximately 0.1. The standard deviation values are approximately 0.1–0.2 and 0.2–0.8 for $f=0.2$ and $0.35$, respectively. The standard deviation values become larger for larger χ*N* values, as presented in Fig. 4.

**Table 4:** Averages and standard deviations of estimated χ*N* for each χ*N* class, which is the same for the teaching images.

	(*f*, *N*) = (0.2, 20)	(*f*, *N*) = (0.2, 40)	(*f*, *N*) = (0.35, 20)	(*f*, *N*) = (0.35, 40)
χ*N* = 26	26.084 ± 0.061	25.850 ± 0.055	26.130 ± 0.239	26.105 ± 0.221
χ*N* = 28	28.081 ± 0.089	27.831 ± 0.069	28.083 ± 0.430	28.113 ± 0.325
χ*N* = 30	30.094 ± 0.100	29.826 ± 0.089	30.097 ± 0.493	30.151 ± 0.370
χ*N* = 32	32.094 ± 0.128	31.804 ± 0.100	32.073 ± 0.591	32.146 ± 0.439
χ*N* = 34	34.120 ± 0.149	33.812 ± 0.118	34.100 ± 0.708	34.186 ± 0.479
χ*N* = 36	36.139 ± 0.190	35.768 ± 0.139	36.139 ± 0.795	36.219 ± 0.544
χ*N* = 38	38.139 ± 0.251	37.837 ± 0.182	37.776 ± 0.678	38.120 ± 0.561
χ*N* = 40	40.127 ± 0.227	39.732 ± 0.183	39.883 ± 0.594	39.926 ± 0.510

**Figure 5:**
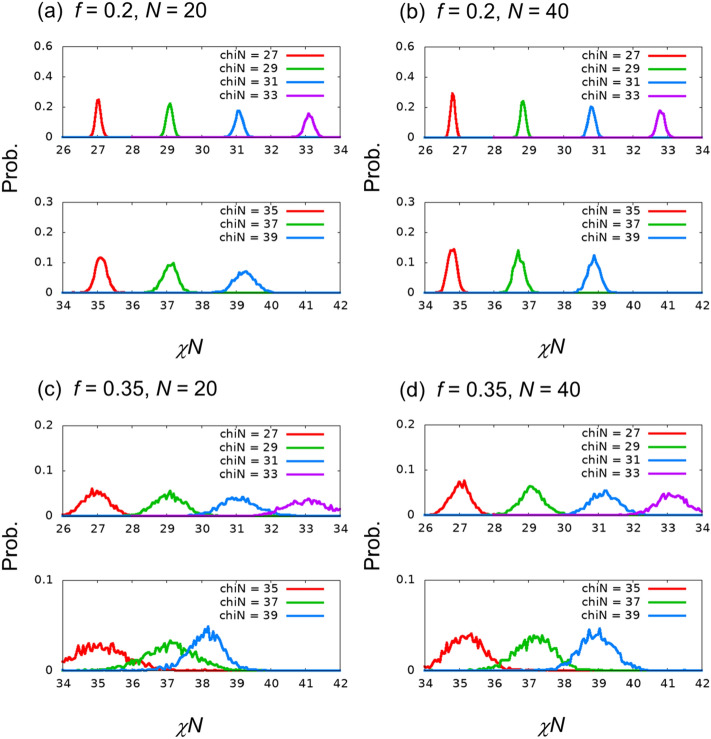
Probability distribution functions of the estimated χ*N* for evaluation data generated with unlearned χ*N* values.

Figure 5 and Table 5 present the distribution, average, and standard deviation values of the estimated χ*N* for independent images of unlearned χ*N,* which are different from those of the training images. We find that the average of estimated χ*N* for the images with $\chi N=39$ for $(f,N)=(0.35, 20)$ differs from the true χ*N* values of the images. The difference from the true value is approximately 0.9. The other estimations are found to be as accurate as the estimations for independent images in the same χ*N* class as the training images. These results indicate that superior regression estimation is possible for $f=0.2$. For $f=0.35$, the error is relatively large, but regression estimation is possible, except for $\chi N>38$ when $(f,N)=(0.35, 20)$. For a detailed investigation on large χ*N*, see Section S5 of the Supplementary Information, which presents the results of the regression for $\chi N=36.5, 37.5, 38.5,$ and 39.5 and the image classification of the 3-class problem with $\chi N=38, 39,$ and 40.

**Table 5:** Averages and standard deviations of estimated χ*N* values for each unlearned χ*N*.

	(*f*, *N*) = (0.2, 20)	(*f*, *N*) = (0.2, 40)	(*f*, *N*) = (0.35, 20)	(*f*, *N*) = (0.35, 40)
χ*N* = 27	27.032 ± 0.081	26.818 ± 0.063	26.976 ± 0.379	27.029 ± 0.293
χ*N* = 29	29.087 ± 0.092	28.843 ± 0.079	29.088 ± 0.457	29.101 ± 0.348
χ*N* = 31	31.090 ± 0.120	30.824 ± 0.095	31.093 ± 0.560	31.189 ± 0.410
χ*N* = 33	33.114 ± 0.138	32.821 ± 0.111	33.072 ± 0.626	33.151 ± 0.465
χ*N* = 35	35.112 ± 0.171	34.799 ± 0.130	35.113 ± 0.771	35.187 ± 0.522
χ*N* = 37	37.103 ± 0.216	36.725 ± 0.157	37.076 ± 0.741	37.173 ± 0.565
χ*N* = 39	39.254 ± 0.295	38.885 ± 0.182	38.114 ± 0.529	38.962 ± 0.502

### Cases with long learning times and transfer learning

In some cases, to obtain small MAEs, long learning times (epochs) and/or transfer learning are applied. In this study, we also attempted to perform learning with large epochs and transfer learning. However, both the cases showed overfitting and poor generalization ability. The detailed results are presented in Sections S6–S9 of the Supplementary Information.

These results indicate that it is a realistic solution to use a trained network, wherein overfitting does not occur in the generalization-ability evaluation of the unlearned χ*N*.

### Confirmation for the binarized images

To confirm the effects of interfacial density gradients on the regression problem and feasibility of χ*N* estimation for stained specimens, we investigated the regression performance for binarized images. Tables 6 and 7 present the average and standard deviation values of the estimated χ*N* for each χ*N* class, as detailed in Section S10 of the Supplementary Information. We consider that DL-based χ*N* estimation for binarized images is learning the characteristics of morphology in the binary images without density gradients. The distributions of the estimated χ*N* for the binarized images are much wider than those for the gray-scale images. Absolute differences from the true values of χ*N* are also larger than those for the gray-scale images. Therefore, we conclude that the gray-scale images have essential information for χ*N* estimation. This suggests that χ*N* can be evaluated accurately without using DL if an arithmetic calculation method for estimating χ*N* from a cross-sectional image is developed. However, at present, such a method is unknown; thus, DL is an effective tool.

**Table 6:** Averages and standard deviations of estimated χ*N* for each χ*N* class for the binarized images. Here, the χ*N* class was same value of the teaching image.

	(*f*, *N*) = (0.2, 20)	(*f*, *N*) = (0.2, 40)	(*f*, *N*) = (0.35, 20)	(*f*, *N*) = (0.35, 40)
χ*N* = 26	26.232 ± 0.548	26.409 ± 0.898	27.229 ± 1.462	28.680 ± 1.761
χ*N* = 28	28.241 ± 1.009	28.230 ± 1.238	29.589 ± 2.224	29.274 ± 1.909
χ*N* = 30	30.572 ± 1.511	30.515 ± 1.546	31.547 ± 2.466	29.911 ± 1.955
χ*N* = 32	32.807 ± 1.871	32.750 ± 1.753	33.125 ± 2.342	31.363 ± 2.133
χ*N* = 34	34.746 ± 1.967	34.521 ± 1.750	34.124 ± 2.342	33.286 ± 2.156
χ*N* = 36	36.330 ± 1.873	35.777 ± 1.795	34.864 ± 2.297	35.836 ± 1.832
χ*N* = 38	37.211 ± 1.792	36.928 ± 1.876	35.076 ± 2.220	37.545 ± 1.400
χ*N* = 40	38.005 ± 1.661	38.322 ± 1.871	37.855 ± 2.099	39.451 ± 1.043

**Table 7:** Averages and standard deviations of estimated χ*N* for each unlearned χ*N* class for the binarized images.

	(*f*, *N*) = (0.2, 20)	(*f*, *N*) = (0.2, 40)	(*f*, *N*) = (0.35, 20)	(*f*, *N*) = (0.35, 40)
χ*N* = 27	27.067 ± 0.772	27.261 ± 1.106	28.288 ± 1.946	28.615 ± 1.749
χ*N* = 29	29.386 ± 1.281	29.485 ± 1.441	30.605 ± 2.375	29.143 ± 1.817
χ*N* = 31	31.640 ± 1.672	31.728 ± 1.631	32.512 ± 2.449	30.660 ± 2.126
χ*N* = 33	33.908 ± 1.925	33.801 ± 1.696	33.593 ± 2.352	32.340 ± 2.151
χ*N* = 35	35.363 ± 1.962	35.190 ± 1.774	34.526 ± 2.284	34.552 ± 2.005
χ*N* = 37	36.745 ± 1.855	36.476 ± 1.768	34.956 ± 2.208	36.712 ± 1.668
χ*N* = 39	37.637 ± 1.741	37.577 ± 1.834	35.328 ± 2.101	38.415 ± 1.058

It should be noted that the χ*N* estimation for the binarized image is predictable as an average, although the error is large. In TEM observations of polymer materials, staining such as by OsO4 is currently essential owing to the limited detector ability. The observed images of the stained sample are considered to correspond to the binarized images. The confirmation that the binarized image has a certain estimation ability is useful information in the future analysis of TEM images of the actual materials.

### Comparison with regression with the 4-class training images

**Figure 6:**
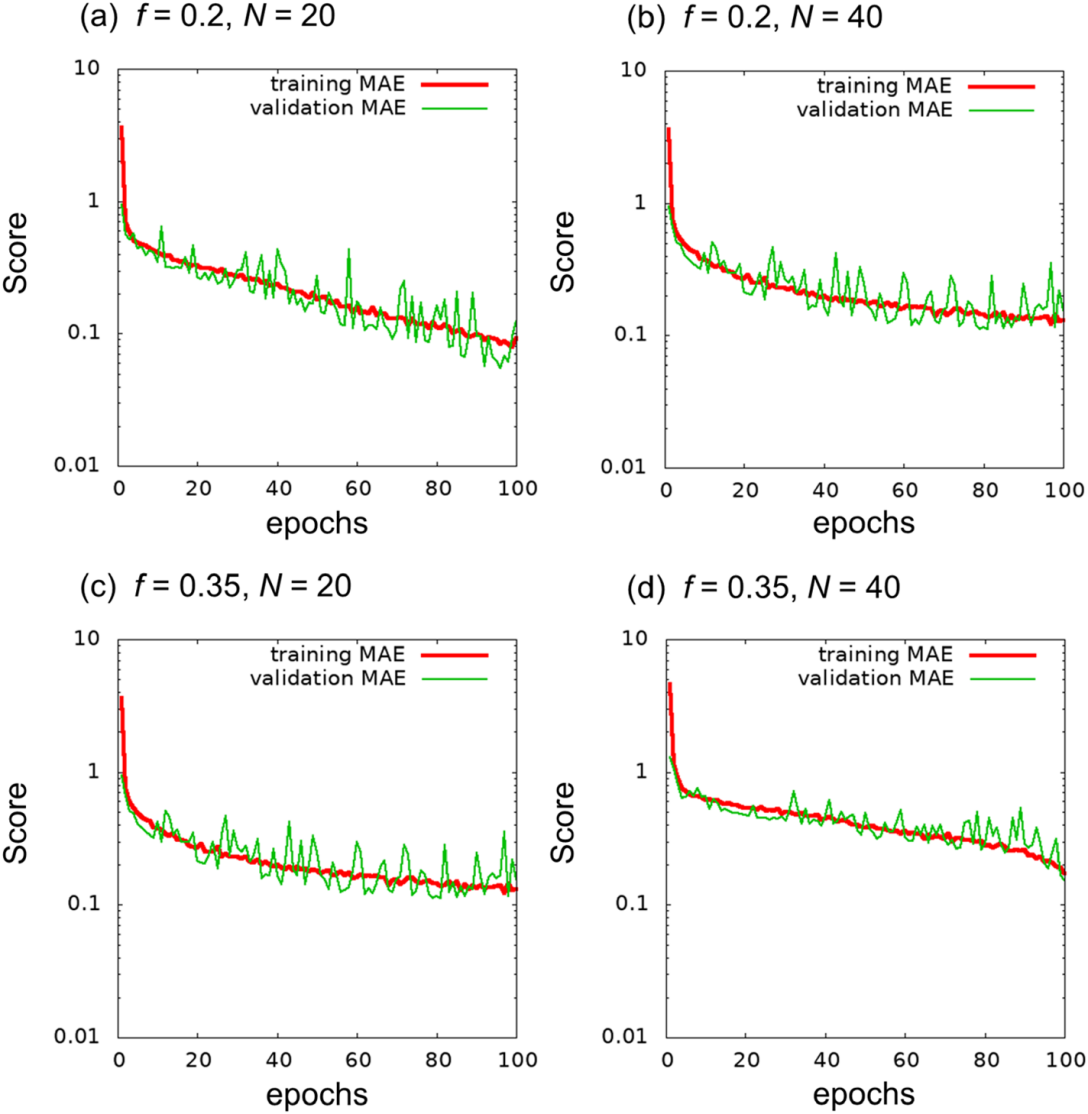
Learning curves of the regression problem with the 4-class teaching images.

To clarify the effects of the number of classes and step size of χ*N* on the error, we investigated the cases of training using the 4-class training images with $\chi N=25, 30, 35,$ and $40$. Figure 6 shows the learning curves for the 4-class training images. The MAEs at 100 epochs in the 4-class problem were smaller than those in the 8-class problem.

**Figure 7:**
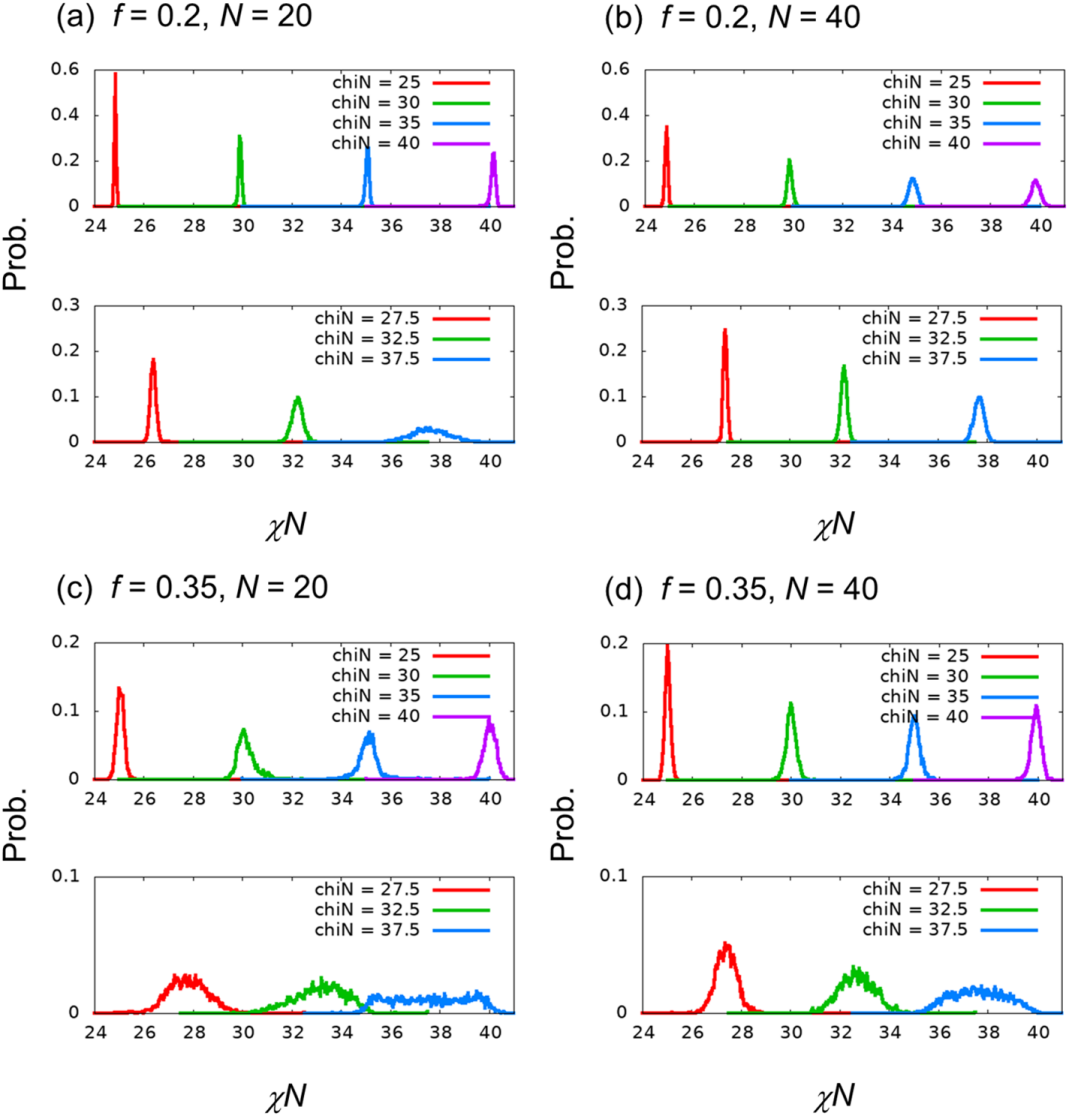
Probability distribution functions of the estimated χ*N* for evaluation data for the 4-class teaching images.

Figure 7 presents the distribution of the estimated χ*N* for the 4-class problem. Except for the cases of $(f,N)=(0.2, 40)$, we find that there is no discriminating ability for $\chi N=37.5$. In particular, for the cases of $(\chi N, f,N)=(37.5 0.35, 20)$, we find sharp peaks at $\chi N=35$ and $40$ which were χ*N* values of the training images as shown in Fig. 7c. These peaks at the χ*N* values of the training data are typical behaviors of overfitting, as observed in Section S7 of the Supplementary Information.

Except for the large χ*N*, it was found that the estimation with the 4-class training images was as accurate as that with the 8-class training images. This finding is supported by the behaviors of the average and standard deviation values presented in Table 8. We consider that this knowledge is useful in the analysis of actual experimental images.

**Table 8:** Averages and standard deviations of estimated χ*N* values for each χ*N* in the 4-class problem*.*

	(*f*, *N*) = (0.2, 20)	(*f*, *N*) = (0.2, 40)	(*f*, *N*) = (0.35, 20)	(*f*, *N*) = (0.35, 40)
χ*N* = 25	24.852 ± 0.032	24.875 ± 0.056	25.062 ± 0.184	25.006 ± 0.111
χ*N* = 30	29.910 ± 0.066	29.876 ± 0.102	30.183 ± 0.436	30.004 ± 0.210
χ*N* = 35	35.064 ± 0.083	34.865 ± 0.163	35.145 ± 0.639	35.011 ± 0.277
χ*N* = 40	40.161 ± 0.104	39.845 ± 0.186	40.004 ± 0.414	39.925 ± 0.270
χ*N* = 27.5	26.385 ± 0.115	27.387 ± 0.079	27.797 ± 0.796	27.401 ± 0.417
χ*N* = 32.5	32.236 ± 0.224	32.201 ± 0.137	33.155 ± 1.031	32.704 ± 0.704
χ*N* = 37.5	37.594 ± 0.727	37.677 ± 0.200	37.563 ± 1.540	37.622 ± 1.071

## Summary and discussion

DL-based methods were studied to estimate the Flory–Huggins χ parameter of A–B diblock copolymers from 2D cross-sectional images generated from SCF calculations, assuming them to observation images from electron microscopes. In this study, we aimed to estimate χ*N* for images created by a particular process. Note that χ*N* estimation, independent of the material processes, cannot be discussed because we used only one image-generation method in this study. Through SCF calculations, 10,000 images for each χ*N* were obtained from cross-sectional views of the 3D phase-separated structures in random directions at randomly selected positions. Here, the 3D density field data were obtained by real-space SCF simulations in the 25–40 χ*N* range for $f=0.2$ and $0.35$ and $N=20$ and $40$. For DL, we used VGG-16^38^.

To show that the generated images can be classified systematically, DL-based image classification was performed. The accuracy for $f=0.2$ was found to be better than that for $f=0.35$ because of the difficulty encountered in distinguishing owing to the resemble images. It was clarified that the accuracy for a larger χ*N* is lower when $f=0.35$.

In addition, we investigated image classification performance of ML with SVM for the histogram of brightness and the HoG features as well as DL for binarized images. The error rates of ML were considerably larger than those of DL. Thus, regression via ML was found to be difficult for these prepared datasets. We also confirmed that the image classification performance by DL for binary images was inferior to those for gray-scale images. The binarization also affected the regression performance. In addition, we found that the DL-based χ*N* estimation for the binarized image was predictable as an average, although the error was large. This is an important finding to extend χ*N* estimation for images in TEM observations of stained polymer materials.

We performed DL of regression problems based on the VGG-16 network model. For the 8-class problem, χ*N* was set at $26, 28, 30, 32, 34, 36, 38,$ and $40$. To evaluate the generalization ability, MAEs for the following two image groups were estimated: (1) independent images generated with the same χ*N* value as that for the training images and (2) independent images with unlearned χ*N* value such as $\chi N=27, 29, 31, 33, 35, 37,$ and $39$.

We investigated the distribution of the estimated χ*N* of independent images with the unlearned χ*N* values. Large χ*N* values could not be accurately estimated, which can be ascribed to the difficulty encountered in image classification. For $(f,N)=(0.35, 20)$, the image classification for the 3-class problem for $\chi N=38, 39,$ and $40$ failed to distinguish images for $\chi N=38$ and $39$. Except when χ*N* was large, we obtained accurate average values of χ*N* for the examined images, and the standard deviation was approximately 0.1 and 0.5 for $f=0.2$ and $0.35$, respectively. To improve the accuracy of estimation for large χ*N*, we require high-resolution images wherein the density gradient at the phase-separation interface can be recognized. Studies in this direction, including experimental observation data, are underway.

Moreover, we found that the learning performances for the 4-class problem were comparable to those for the 8-class problem except when χ*N* was large. This information is useful for the analysis of experimental image data. On the other hand, given that the estimation with the 8-class teacher dataset was more accurate than that with the 4-class dataset, the performance could be improved with the incorporation of smaller χ*N* intervals into the teacher data. For example, it is difficult to prepare specimens that have a wide χ*N* range with 0.1 intervals even in simulations; however, it may not be impossible. Research that provides insights into how small an χ*N* interval is required for more accurate estimations, would be an important next step in this field.

To estimate χ*N* from experimental images, in addition to the effects of binarization associated with observations of stained specimens, we should train a regression network model that is robust to the effects of noise and image adjustments (including focus) of experimental data. To investigate random local noises and variations in image contrast and brightness, a large amount of experimental image data must be analyzed and pseudo image data must be generated accordingly. Recently developed electron-microscope automation techniques can be applied to observe a large area of images from one stained specimen at one observation. Research in these directions is also being conducted.

In this study, we considered images created solely from a particular process of phase separations. This limits our ability to estimate χ*N* only for that specific material process. Although the effectiveness of the learned network was limited to a specific process, we expected the estimation ability of the physical parameters governing phase-separation to be utilizable not only for polymers but also for metals. In the research and development of real materials, χ*N* is expected to be estimated from structures obtained from various material processes. Further prospects in this field include an investigation into the feasibility of χ*N* estimation, independent of material processes.

## Methods

### Image data preparation through SCF calculation

For $f=0.2$ and $0.35$, 3D field data of the phase-separated structure of A-B BCP were obtained using the OCTA/SUSHI package^92,93^ based on the real-space SCF calculation^52,53^. The theoretical background is briefly explained in Section S11 of the Supplementary Information. The system size was set at 128 × 128 × 128 under PBCs, and a regular 128 × 128 × 128 grid mesh was used. In the present study, the following cases were examined:$N=20$ and $40$. According to the mean field prediction^94^, the boundary value, χ*N*, of the order–disorder phase transition is approximately 23.5 for $f=0.2$ and 12.5 for $f=0.35$. Thus, we generated images for $\chi N\ge 25$, as presented in Fig. 1. In practice, we obtained the 3D field data with an χ*N* interval of 0.5.

All the images were obtained from cross-sectional views of the 3D density field data of the A domain in random directions at randomly selected positions. A total of 10,000 input images with 64 × 64 pixels were prepared for each class. In generating a cross-sectional view with 64 × 64 pixels from 3D field data of 128 × 128 × 128 grids under the PBCs, we performed linear-weight interpolation. Here, the images were 8-bit gray-scale images. We placed the same data in 3 RGB channels for generality in preliminarily tests such as transfer learning using the weight data trained by ImageNet^38^. Learning with the same data on three channels did not have any significant effect, except for a slight difference in convergence behaviors.

This differs from the method of obtaining highly symmetric structures from SCF calculations. To construct ordered structures in the shape of lamellae, cylinders, and gyroids, artificial initial estimate and cell-size optimization—a parameter search of the box size to minimize the free energy^95^—are effective. The initial value and search range are important to obtain a reasonable solution. If we start from uniformly mixed initial states, hydrodynamic effects are essential to obtain ordered structures^101^. By contrast, to obtain random network of phase-separated domains, spatially uncorrelated fields were used as initial density profiles and cell-size optimization was not used.

### Image classification by DL

**Figure 8:**
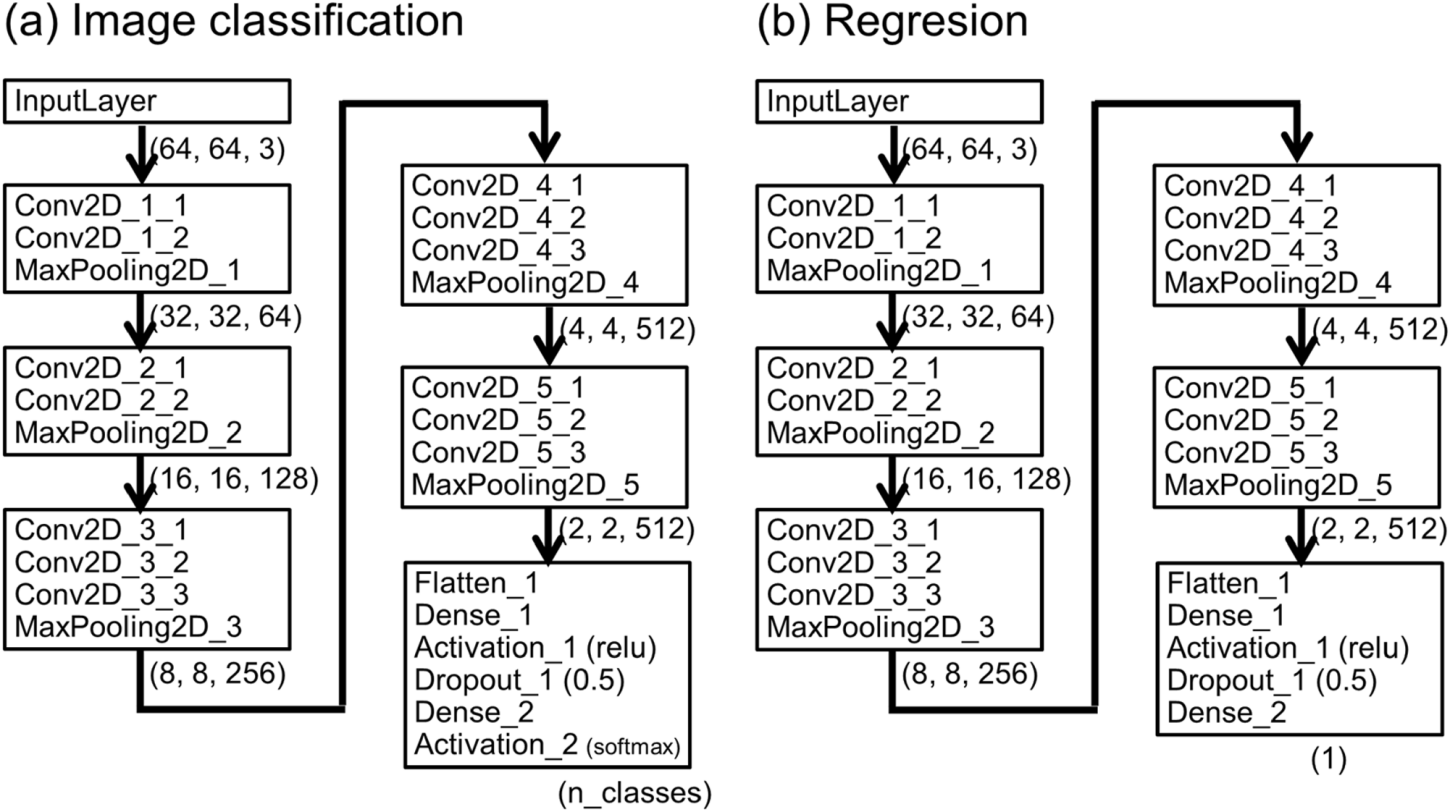
Schematic images of network architectures for (**a**) image-classification problem and (**b**) regression problem based on the VGG-16 model. The first five convolutional blocks are the same, but the last block is different. For the image-classification problem, the output is a vector whose number of elements equals the number of classes. For the regression problem, the output is a scalar. Here, the numbers at the lower right of each block denote the number of elements of the output tensor of each block.

In the image-classification problem, the labels of the training images are learned and the trained model outputs the estimated probability of each label for an arbitrary image. CNNs are well known to have high image-classification ability^38–44^. Keras^96^ provides all popular network models for image classification including VGG-16 model, which is one of the more successful CNN models. Comparisons among popular network models provided by Keras^96^ are presented in Section S12 of the Supplementary Information. The VGG-16 model has 16 layers, including five convolutional blocks (13 convolutional layers), as shown in Fig. 8.

To determine the parameters of the VGG-16 model, we used TensorFlow^97^ as the backend for Keras. For image classification, 6000 and 2000 images per χ*N* class were used as training and testing images, respectively, for the learning and for evaluating the generalization ability. The stochastic gradient descent (SGD) method was used as the optimizer for the classification problem; a standard learning rate of 10^–4^ and momentum 0.9 was used for simplicity.

### Estimation (regression) of the Flory–Huggins parameter via DL

In the regression problem, the values of the training images are learned and the trained model outputs the estimated values for an arbitrary image. For the regression problem, we used a network based on the VGG-16 model, as presented in Fig. 8b. Compared to the classification problem, in the regression problem, the last block is different, as shown in Fig. 8. In the regression problem, we used the adaptive moment estimation (Adam)^102^ as the optimizer, with a standard learning rate of 10^–6^ and $\left({\beta }_{1},{\beta }_{2}\right)=(0.9, 0.999)$ for simplicity.

## Supplementary Information

Supplementary Information.

## Data Availability

All generated image data used are available from the corresponding author upon reasonable request.
