# Supplementary material for: Deep learning-based estimation of Flory–Huggins parameter of A–B block copolymers from cross-sectional images of phase-separated structures
Source: Sci Rep. 2021 Jun 10;11:12322. doi: 10.1038/s41598-021-91761-8 (PMC8192782; doi:10.1038/s41598-021-91761-8)
Supplement: Supplementary file 1 — Supplementary Information. [file 41598_2021_91761_MOESM1_ESM.pdf]

Supplementary Information

Deep Learning-Based Estimation of Flory-Huggins Parameter of A-B Block Copolymers from Cross-Sectional Images of Phase-Separated Structures

Katsumi Hagita*[1], Takeshi Aoyagi[2], Yuto Abe[1], Shinya Genda[1], Takashi Honda[3]

[1]Department of Applied Physics, National Defense Academy, 1-10-20 Hashirimizu, Yokosuka 239-8686, Japan

[2]Research Center for Computational Design of Advanced Functional Materials, National Institute of Advanced Industrial Science and Technology, Central 2, 1-1-1, Umezono, Tsukuba, Ibaraki 305-8568, Japan

[3]Zeon Corporation, 1-2-1 Yako, Kawasaki-ku, Kawasaki 210-9507, Japan

S1. Image classifications of the 6- and 8-class problems

Confusion matrices and learning curves for the 6-class problem are illustrated in Table S1 and Fig. S1, respectively. The error rates E were 0.0, 0.0, 4.0×10^{-3}, and 1.98×10^{-2} for (f, N) = (0.2, 20), (0.2, 40), (0.35, 20), and (0.35, 40), respectively. For the 6-class problem, the probability that the images with $f = 0.35$ were more difficult to learn compared to $f = 0.2$ and this was also observed in both 4- and 8-class problems. We found that these behaviors of E were consistent with the behavior of the learning curves presented in Fig. S1.

Table S1. Confusion matrices of the 6-class problem at 100 epochs.

(a) $f = 0.2$, $N = 20$		VGG-16 estimated χN class					
		25	28	31	34	37	40
Actual	$\chi N = 25$	2000	0	0	0	0	0
	$\chi N = 28$	0	2000	0	0	0	0
	$\chi N = 31$	0	0	2000	0	0	0
	$\chi N = 34$	0	0	0	2000	0	0
	$\chi N = 37$	0	0	0	0	2000	0
	$\chi N = 40$	0	0	0	0	0	2000

(b) $f = 0.2$, $N = 40$		VGG-16 estimated χN class					
		25	28	31	34	37	40
Actual	$\chi N = 25$	2000	0	0	0	0	0
	$\chi N = 28$	0	2000	0	0	0	0
	$\chi N = 31$	0	0	2000	0	0	0
	$\chi N = 34$	0	0	0	2000	0	0
	$\chi N = 37$	0	0	0	0	2000	0
	$\chi N = 40$	0	0	0	0	0	2000

(c) $f = 0.35$, $N = 20$		VGG-16 estimated χN class					
		25	28	31	34	37	40
Actual	$\chi N = 25$	1998	2	0	0	0	0
	$\chi N = 28$	0	1994	6	0	0	0
	$\chi N = 31$	0	0	1988	12	0	0
	$\chi N = 34$	0	0	2	1987	11	0
	$\chi N = 37$	0	0	0	6	1990	4
	$\chi N = 40$	0	0	0	0	5	1995

(d) $f = 0.35$, $N = 40$		VGG-16 estimated χN class					
		25	28	31	34	37	40
Actual	$\chi N = 25$	1998	2	0	0	0	0
	$\chi N = 28$	3	1990	7	0	0	0
	$\chi N = 31$	0	60	1976	17	1	0
	$\chi N = 34$	0	0	13	1896	87	4
	$\chi N = 37$	0	0	2	13	1915	70
	$\chi N = 40$	0	0	1	2	10	1987

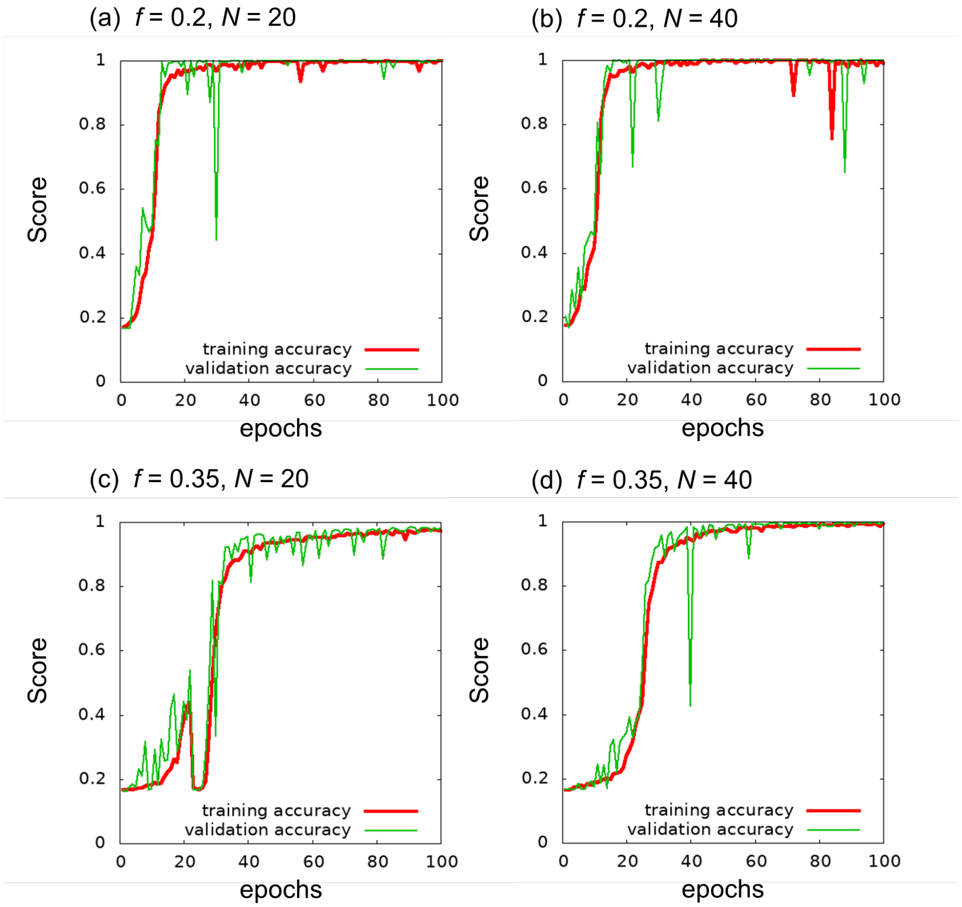

Figure S1. Learning curves of the 6-class image classification under training until 100 epochs.

Table S2 lists the confusion matrices of the 8-class problem at 100 epochs. The error rates E were 2.5×10^{-4}, 4.94×10^{-3}, 1.46×10^{-2}, and 1.09×10^{-2} for (f, N) = (0.2, 20), (0.2, 40), (0.35, 20), and (0.35, 40), respectively. For the 8-class problem, the probability that the images for $f = 0.35$ were more difficult to learn compared to $f = 0.2$ and this was also observed in 4-class problem. We found that these behaviors of E were consistent with the behavior of the learning curves presented in Fig. S2.

Table S2. Confusion matrices of the 8-class problem at 100 epochs.

(a) $f = 0.2$, $N = 20$		VGG-16 estimated χN class							
		26	28	30	32	34	36	38	40
Actual	$\chi N = 26$	2000	0	0	0	0	0	0	0
	$\chi N = 28$	0	2000	0	0	0	0	0	0
	$\chi N = 30$	0	0	2000	0	0	0	0	0
	$\chi N = 32$	0	0	0	2000	0	0	0	0
	$\chi N = 34$	0	0	0	0	2000	0	0	0
	$\chi N = 36$	0	0	0	0	0	2000	0	0
	$\chi N = 38$	0	0	0	0	0	0	1997	3
	$\chi N = 40$	0	0	0	0	0	0	1	1999

(b) $f = 0.2$, $N = 40$		VGG-16 estimated χN class							
		26	28	30	32	34	36	38	40
Actual	$\chi N = 26$	2000	0	0	0	0	0	0	0
	$\chi N = 28$	0	2000	0	0	0	0	0	0
	$\chi N = 30$	0	0	2000	0	0	0	0	0
	$\chi N = 32$	0	0	0	2000	0	0	0	0
	$\chi N = 34$	0	0	0	0	2000	0	0	0
	$\chi N = 36$	0	0	0	0	0	2000	0	0
	$\chi N = 38$	0	0	0	0	0	19	1981	0
	$\chi N = 40$	0	0	0	0	0	0	60	1940

(c) $f = 0.35$, $N = 20$		VGG-16 estimated χN class							
		26	28	30	32	34	36	38	40
Actual	$\chi N = 26$	1999	1	0	0	0	0	0	0
	$\chi N = 28$	5	1993	2	0	0	0	0	0
	$\chi N = 30$	0	3	1990	7	0	0	0	0
	$\chi N = 32$	0	0	3	1980	16	1	0	0
	$\chi N = 34$	0	0	0	15	1951	33	1	0
	$\chi N = 36$	0	0	0	1	8	1919	72	0
	$\chi N = 38$	0	0	0	0	2	33	1965	0
	$\chi N = 40$	0	0	0	1	2	12	15	1970

(d) $f = 0.35$, $N = 40$		VGG-16 estimated χN class							
		26	28	30	32	34	36	38	40
Actual	$\chi N = 26$	1994	6	0	0	0	0	0	0
	$\chi N = 28$	1	1971	28	0	0	0	0	0
	$\chi N = 30$	0	1	1983	16	0	0	0	0
	$\chi N = 32$	0	0	4	1967	29	0	0	0
	$\chi N = 34$	0	0	0	2	1987	11	0	0
	$\chi N = 36$	0	0	0	0	9	1958	33	0
	$\chi N = 38$	0	0	0	0	0	13	1981	6
	$\chi N = 40$	0	0	0	0	0	0	16	1984

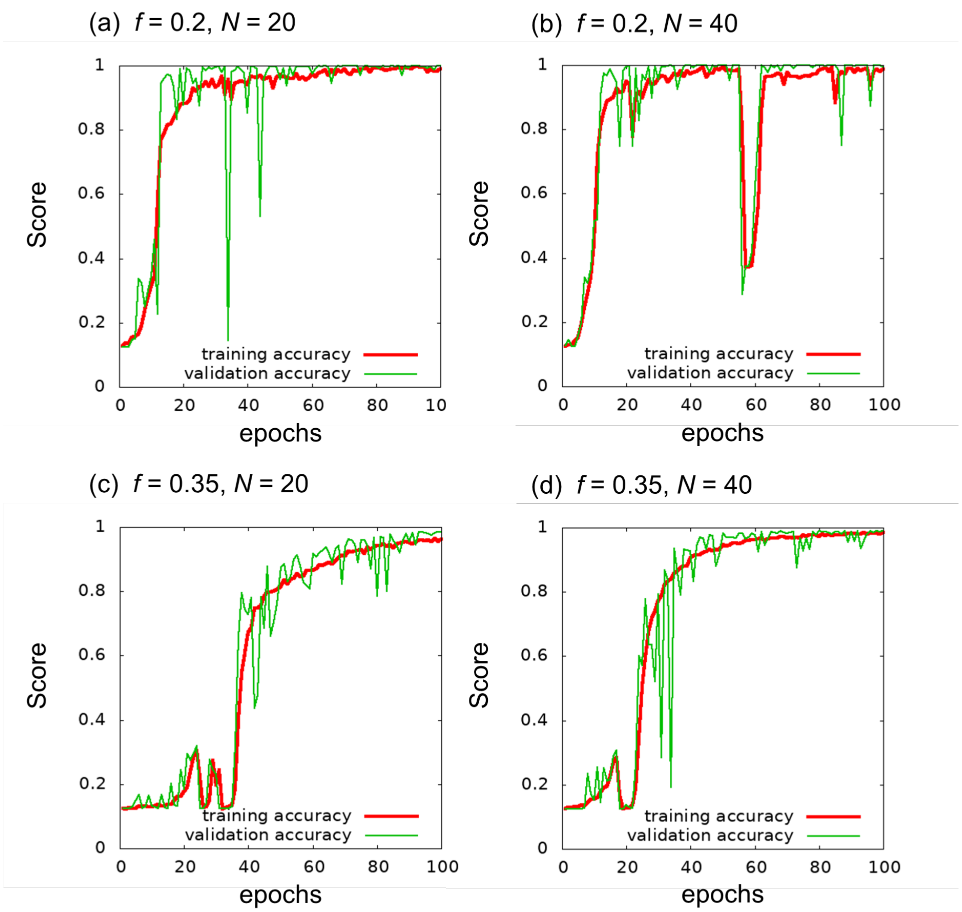

Figure S2. Learning curves of the 8-class image classification under training until 100 epochs.

For verification, we performed two additional independent learning steps for each (f, N). When (f, N) = (0.35, 20), we encountered a learning failure case, as shown in Table S3 (a). As can be observed in Fig. S3 (a), the reason for this failure was that the trained network at 100 epochs accidentally had a significantly low validation accuracy. In the third training, the errors in Table S3 (b), was smaller than those of the second training in Table S3 (a). This behavior was reasonable based on the behavior of the learning curve presented in Fig. S3 (b).

Table S3. Confusion matrices of the 8-class problem for (f, N) = (0.35, 20) at 100 epochs.

(a) 2nd training of $f = 0.35$, $N = 20$		VGG-16 estimated χN class							
		26	28	30	32	34	36	38	40
Actual	$\chi N = 26$	1716	284	0	0	0	0	0	0
	$\chi N = 28$	0	1326	674	0	0	0	0	0
	$\chi N = 30$	0	0	610	1390	0	0	0	0
	$\chi N = 32$	0	0	0	178	1821	1	0	0
	$\chi N = 34$	0	0	0	0	68	1880	12	40
	$\chi N = 36$	0	0	0	0	0	16	1927	57
	$\chi N = 38$	0	0	0	0	0	0	1989	11
	$\chi N = 40$	0	0	0	0	0	0	0	2000

(b) 3rd training of $f = 0.35$, $N = 20$		VGG-16 estimated χN class							
		26	28	30	32	34	36	38	40
Actual	$\chi N = 26$	1989	11	0	0	0	0	0	0
	$\chi N = 28$	1	1994	5	0	0	0	0	0
	$\chi N = 30$	0	1	1991	8	0	0	0	0
	$\chi N = 32$	0	0	3	1963	33	1	0	0
	$\chi N = 34$	0	0	0	12	1932	54	2	0
	$\chi N = 36$	0	0	0	0	10	1849	141	0
	$\chi N = 38$	0	0	0	0	3	18	1979	0
	$\chi N = 40$	0	0	0	1	2	6	11	1980

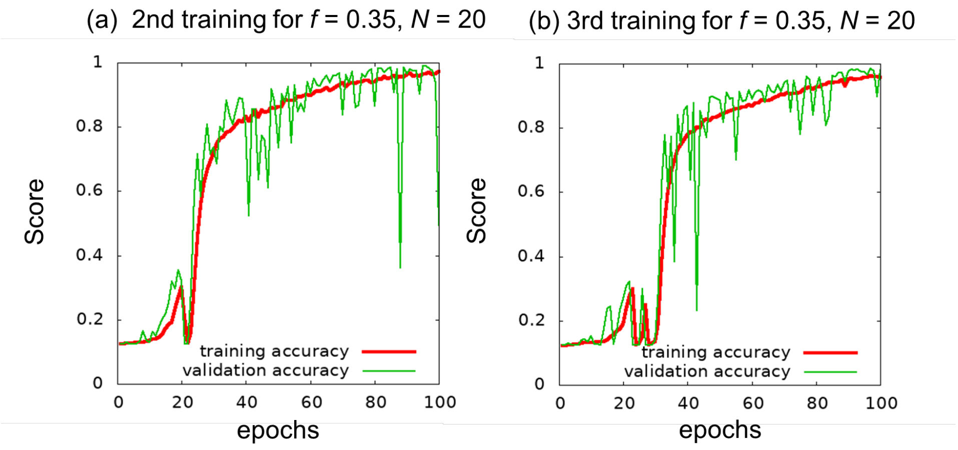

Figure S3. Learning curves of the 8-class image classification for (f, N) = (0.35, 20) under training until 100 epochs.

S2. Confusion matrixes of image classifications obtained from ML and DL for the 4-class problem

Tables S4–S6 present the confusion matrices of image classifications for the 4-class problem using ML with SVM for the histogram of brightness and the HoG features, and DL for binarized images. The overall performance of each method is listed in Table 2. From Table S4 (a) and (b), it can be observed that for f = 0.2, ML with SVM for the histogram of brightness failed clearly. From Table S4 (c) and (d), it can be observed that for f = 0.35, ML with the histogram of brightness were able to classify small χN. As shown in Table S5, for ML with HoG features, the error was large. These behaviors of ML were similar to those of the 8-class problem, which is discussed in the next section. When DL was used for binary images, many classes were incorrectly classified as adjacent classes. These behaviors are similar to the 8-class problem.

Table S4. Confusion matrices of the 4-class problem via ML with SVM for histogram of brightness.

(a) f = 0.2, N = 20		estimated χN class			
		25	30	35	40
Actual	χN = 25	0	0	2000	0
	χN = 30	0	0	2000	0
	χN = 35	0	0	2000	0
	χN = 40	0	0	198	1802
(b) f = 0.2, N = 40		estimated χN class			
		25	30	35	40
Actual	χN = 25	0	0	2000	0
	χN = 30	0	0	2000	0
	χN = 35	0	0	2000	0
	χN = 40	0	0	93	1907
(c) f = 0.35, N = 20		estimated χN class			
		25	30	35	40
Actual	χN = 25	2000	0	0	0
	χN = 30	21	1979	0	0
	χN = 35	0	0	1387	613
	χN = 40	0	1	563	1436
(d) f = 0.35, N = 40		estimated χN class			
		25	30	35	40
Actual	χN = 25	2000	0	0	0
	χN = 30	0	1986	13	1
	χN = 35	0	23	878	1099
	χN = 40	0	19	878	1103

Table S5. Confusion matrices of the 4-class problem via ML with SVM for HoG features.

(a) $f = 0.2$, $N = 20$		estimated χN class			
		25	30	35	40
Actual	$\chi N = 25$	1493	442	47	18
	$\chi N = 30$	282	935	415	368
	$\chi N = 35$	72	601	606	721
	$\chi N = 40$	33	472	622	873
(b) $f = 0.2$, $N = 40$		estimated χN class			
		25	30	35	40
Actual	$\chi N = 25$	375	687	579	359
	$\chi N = 30$	373	721	604	302
	$\chi N = 35$	442	731	531	296
	$\chi N = 40$	459	661	489	391
(c) $f = 0.35$, $N = 20$		estimated χN class			
		25	30	35	40
Actual	$\chi N = 25$	1236	329	329	106
	$\chi N = 30$	644	495	532	329
	$\chi N = 35$	478	503	545	474
	$\chi N = 40$	58	253	324	1365
(d) $f = 0.35$, $N = 40$		estimated χN class			
		25	30	35	40
Actual	$\chi N = 25$	883	596	317	204
	$\chi N = 30$	816	574	338	272
	$\chi N = 35$	515	458	460	567
	$\chi N = 40$	195	208	412	1185

Table S6. Confusion matrices of DL-based image classification for the 4-class problem of binarized images at 100 epochs.

(a) $f = 0.2$, $N = 20$		estimated χN class			
		25	30	35	40
Actual	$\chi N = 25$	1995	5	0	0
	$\chi N = 30$	0	1804	196	0
	$\chi N = 35$	0	42	1355	603
	$\chi N = 40$	0	0	224	1776
(b) $f = 0.2$, $N = 40$		estimated χN class			
		25	30	35	40
Actual	$\chi N = 25$	1994	6	0	0
	$\chi N = 30$	3	1748	248	1
	$\chi N = 35$	0	27	1241	732
	$\chi N = 40$	0	0	77	1923
(c) $f = 0.35$, $N = 20$		estimated χN class			
		25	30	35	40
Actual	$\chi N = 25$	1956	44	0	0
	$\chi N = 30$	119	1405	474	2
	$\chi N = 35$	4	542	1404	50
	$\chi N = 40$	0	1	108	1891
(d) $f = 0.35$, $N = 40$		estimated χN class			
		25	30	35	40
Actual	$\chi N = 25$	1516	478	6	0
	$\chi N = 30$	777	1080	143	0
	$\chi N = 35$	20	373	1605	2
	$\chi N = 40$	0	1	208	1791

S3. Confusion matrices of image classifications by ML and DL for the 8-class problem

Tables S7 and S8 shows confusion matrices of image classifications for the 8-class problem obtained using ML with SVM for the histogram of brightness and HoG features. The overall performance of each method is listed in Table 3. The behaviors of the obtained results were similar to those of the 4-class problem discussed in the previous section S2. Table S9 shows the confusion matrices of image classifications for the 8-class problem obtained using DL for binarized images. The overall performance is listed in Table 3. The behaviors of the obtained results were similar to those of the 4-class problem discussed in the previous section.

Table S7. Confusion matrices of the 8-class problem via ML with histogram of brightness

(a) f = 0.2, N = 20		estimated χN class							
		26	28	30	32	34	36	38	40
Actual	$\chi N = 26$	0	0	0	0	0	0	2000	0
	$\chi N = 28$	0	0	0	0	0	0	2000	0
	$\chi N = 30$	0	0	0	0	0	0	2000	0
	$\chi N = 32$	0	0	0	0	0	0	2000	0
	$\chi N = 34$	0	0	0	0	0	0	2000	0
	$\chi N = 36$	0	0	0	0	0	0	2000	0
	$\chi N = 38$	0	0	0	0	0	0	2000	0
	$\chi N = 40$	0	0	0	0	0	0	205	1795
(b) f = 0.2, N = 40		estimated χN class							
		26	28	30	32	34	36	38	40
Actual	$\chi N = 26$	0	0	0	0	0	0	2000	0
	$\chi N = 28$	0	0	0	0	0	0	2000	0
	$\chi N = 30$	0	0	0	0	0	0	2000	0
	$\chi N = 32$	0	0	0	0	0	0	2000	0
	$\chi N = 34$	0	0	0	0	0	0	2000	0
	$\chi N = 36$	0	0	0	0	0	0	2000	0
	$\chi N = 38$	0	0	0	0	0	0	2000	0
	$\chi N = 40$	0	0	0	0	0	116	0	1884
(c) f = 0.35, N = 20		estimated χN class							
		26	28	30	32	34	36	38	40
Actual	$\chi N = 26$	1994	6	0	0	0	0	0	0
	$\chi N = 28$	1143	788	69	0	0	0	0	0
	$\chi N = 30$	22	268	1628	82	0	0	0	0
	$\chi N = 32$	0	0	162	1732	106	0	0	0
	$\chi N = 34$	0	0	0	130	1003	158	0	709
	$\chi N = 36$	0	0	0	0	16	1494	261	229
	$\chi N = 38$	0	0	0	0	0	282	1716	2
	$\chi N = 40$	0	0	0	66	835	231	0	868
(d) f = 0.35, N = 40		estimated χN class							
		26	28	30	32	34	36	38	40
Actual	$\chi N = 26$	1998	2	0	0	0	0	0	0
	$\chi N = 28$	0	1972	28	0	0	0	0	0
	$\chi N = 30$	0	24	1801	174	0	0	0	0
	$\chi N = 32$	0	0	192	1318	397	9	13	71
	$\chi N = 34$	0	0	0	449	768	112	235	430
	$\chi N = 36$	0	0	0	96	363	183	857	501
	$\chi N = 38$	0	0	0	30	195	141	1290	344
	$\chi N = 40$	0	0	0	156	520	157	682	485

Table S8. Confusion matrices of the 8-class problem via ML with SVM for HoG features

(a) $f = 0.2$, $N = 20$		estimated χN class							
		26	28	30	32	34	36	38	40
Actual	$\chi N = 26$	1535	379	71	12	0	2	0	1
	$\chi N = 28$	601	771	347	123	71	41	22	24
	$\chi N = 30$	138	524	453	270	214	143	121	137
	$\chi N = 32$	51	298	376	291	300	254	224	206
	$\chi N = 34$	22	182	297	319	277	333	268	302
	$\chi N = 36$	12	135	242	280	336	326	330	339
	$\chi N = 38$	8	96	198	243	342	366	369	378
	$\chi N = 40$	7	62	165	208	316	386	389	467
(b) $f = 0.2$, $N = 40$		estimated χN class							
		26	28	30	32	34	36	38	40
Actual	$\chi N = 26$	1517	390	68	15	3	4	2	1
	$\chi N = 28$	604	726	374	141	59	46	25	25
	$\chi N = 30$	164	492	456	264	190	166	141	127
	$\chi N = 32$	56	291	390	310	298	249	183	223
	$\chi N = 34$	24	188	313	305	293	292	251	334
	$\chi N = 36$	15	130	251	253	331	318	316	386
	$\chi N = 38$	8	88	237	212	343	331	359	422
	$\chi N = 40$	5	63	189	195	334	334	392	488
(c) $f = 0.35$, $N = 20$		estimated χN class							
		26	28	30	32	34	36	38	40
Actual	$\chi N = 26$	711	314	205	187	152	149	162	120
	$\chi N = 28$	501	265	223	230	182	185	164	250
	$\chi N = 30$	459	260	237	211	169	192	172	300
	$\chi N = 32$	354	253	202	219	202	213	185	372
	$\chi N = 34$	351	232	192	207	216	206	182	414
	$\chi N = 36$	310	225	184	245	188	210	214	424
	$\chi N = 38$	317	215	182	240	200	228	198	420
	$\chi N = 40$	41	61	76	134	108	142	151	1287
(d) $f = 0.35$, $N = 40$		estimated χN class							
		26	28	30	32	34	36	38	40
Actual	$\chi N = 26$	584	380	263	201	152	143	121	156
	$\chi N = 28$	538	364	264	214	176	119	153	172
	$\chi N = 30$	494	354	273	208	168	161	165	177
	$\chi N = 32$	444	311	281	224	177	168	185	210
	$\chi N = 34$	323	271	239	192	195	192	244	344
	$\chi N = 36$	223	168	195	177	242	231	281	483
	$\chi N = 38$	162	154	145	141	208	210	339	641
	$\chi N = 40$	100	103	113	102	139	214	313	916

Table S9. Confusion matrices of the 8-class problem for binarized images.

(a) $f = 0.2$, $N = 20$		estimated χN class							
		26	28	30	32	34	36	38	40
Actual	$\chi N = 26$	1980	20	0	0	0	0	0	0
	$\chi N = 28$	185	1762	53	0	0	0	0	0
	$\chi N = 30$	0	578	1207	206	0	0	0	0
	$\chi N = 32$	0	30	611	1104	237	17	1	0
	$\chi N = 34$	0	1	98	761	803	251	73	13
	$\chi N = 36$	0	0	11	246	738	521	343	141
	$\chi N = 38$	0	0	0	84	406	547	538	425
	$\chi N = 40$	0	0	0	16	200	391	584	809
(b) $f = 0.2$, $N = 40$		estimated χN class							
		26	28	30	32	34	36	38	40
Actual	$\chi N = 26$	1443	557	0	0	0	0	0	0
	$\chi N = 28$	26	1385	585	0	0	0	0	0
	$\chi N = 30$	1	94	1353	543	0	0	0	0
	$\chi N = 32$	0	3	265	1300	392	35	5	0
	$\chi N = 34$	0	0	26	596	911	338	76	3
	$\chi N = 36$	0	0	1	140	634	775	391	59
	$\chi N = 38$	0	0	0	25	229	642	825	279
	$\chi N = 40$	0	0	0	1	34	240	785	940
(c) $f = 0.35$, $N = 20$		estimated χN class							
		26	28	30	32	34	36	38	40
Actual	$\chi N = 26$	1648	339	13	0	0	0	0	0
	$\chi N = 28$	447	1074	394	77	5	1	2	0
	$\chi N = 30$	48	578	733	425	85	98	33	0
	$\chi N = 32$	3	140	501	650	170	317	216	3
	$\chi N = 34$	2	40	282	525	191	418	535	7
	$\chi N = 36$	0	13	108	329	163	469	916	2
	$\chi N = 38$	0	6	66	259	105	422	1139	3
	$\chi N = 40$	0	3	11	40	44	6	47	1849
(d) $f = 0.35$, $N = 40$		estimated χN class							
		26	28	30	32	34	36	38	40
Actual	$\chi N = 26$	1025	447	345	163	20	0	0	0
	$\chi N = 28$	765	411	465	293	64	2	0	0
	$\chi N = 30$	445	400	556	475	112	12	0	0
	$\chi N = 32$	136	211	404	702	478	69	0	0
	$\chi N = 34$	10	33	148	486	827	448	47	1
	$\chi N = 36$	1	1	6	70	431	981	496	14
	$\chi N = 38$	0	0	0	4	52	455	1365	124
	$\chi N = 40$	0	0	0	0	3	5	106	1886

S4. Regression problem with 50 epochs

For verification, we examined the regression problem with 50 epochs instead of 100 epochs in the main text (Figs. 3-5 and Tables 4 and 5). Figure S4 shows the learning curves for 50 epochs. This run was an independent run from the training for Figs. 3-5 and Tables 4 and 5 in the main text. We found that the results obtained were similar to those obtained for 100 epochs.

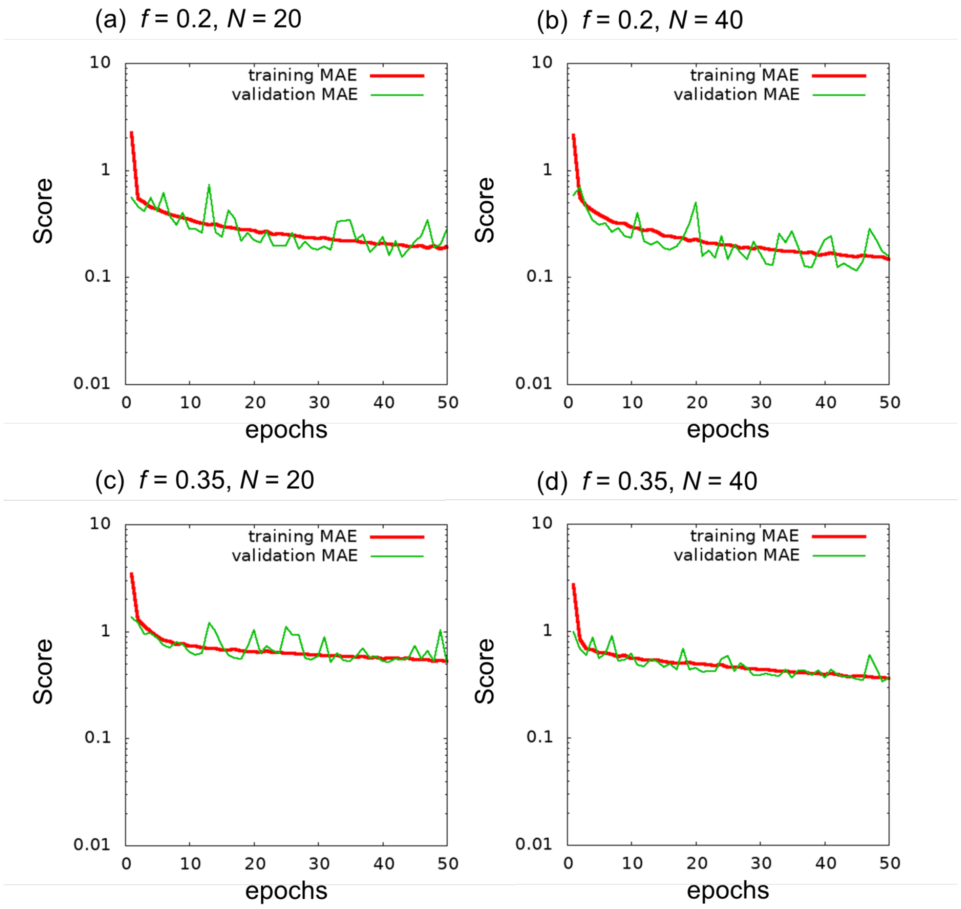

Figure S4. Learning curves of the regression problem until 50 epochs.

Table S10. Averages and standard deviations of estimated χN for each χN class, which was same value of the teaching image.

	$(f, N) = (0.2, 20)$	$(f, N) = (0.2, 40)$	$(f, N) = (0.35, 20)$	$(f, N) = (0.35, 40)$
$\chi N = 26$	26.198 ± 0.087	25.947 ± 0.066	25.860 ± 0.251	25.944 ± 0.241
$\chi N = 28$	28.208 ± 0.110	27.915 ± 0.080	27.747 ± 0.464	27.847 ± 0.324
$\chi N = 30$	30.252 ± 0.129	29.908 ± 0.105	29.792 ± 0.535	29.842 ± 0.373
$\chi N = 32$	32.260 ± 0.153	31.885 ± 0.116	31.776 ± 0.634	31.816 ± 0.443
$\chi N = 34$	34.325 ± 0.186	33.858 ± 0.134	33.785 ± 0.781	33.847 ± 0.464
$\chi N = 36$	36.337 ± 0.234	35.809 ± 0.161	35.809 ± 0.868	35.875 ± 0.535
$\chi N = 38$	38.366 ± 0.299	37.890 ± 0.200	37.567 ± 0.775	37.830 ± 0.571
$\chi N = 40$	40.149 ± 0.278	39.731 ± 0.215	39.571 ± 0.774	39.886 ± 0.566

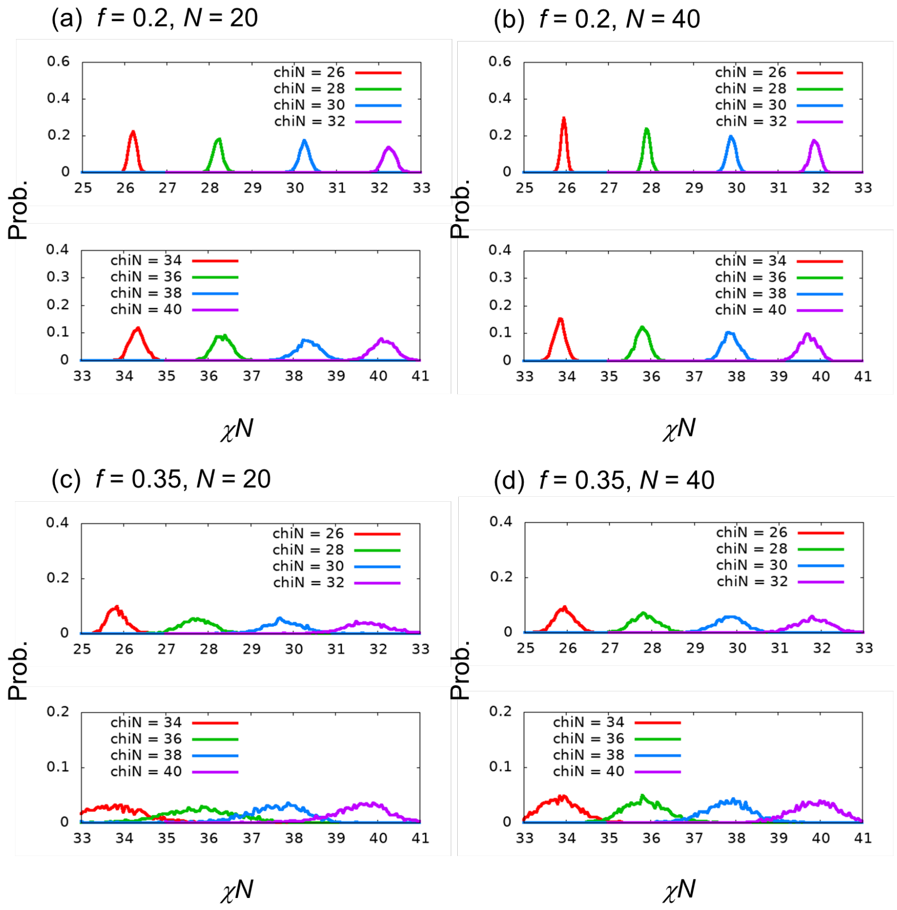

Figure S5. Probability distribution functions of the estimated χN for evaluation data with the same χN values for the training with 50 epochs. Here, the size of each bin was 0.05.

Table S11. Averages and standard deviations of estimated χN for each unlearned χN class.

	$(f, N) = (0.2, 20)$	$(f, N) = (0.2, 40)$	$(f, N) = (0.35, 20)$	$(f, N) = (0.35, 40)$
$\chi N = 27$	27.173 ± 0.097	26.910 ± 0.075	26.668 ± 0.382	26.832 ± 0.288
$\chi N = 29$	29.226 ± 0.116	28.921 ± 0.092	27.772 ± 0.497	27.792 ± 0.354
$\chi N = 31$	31.255 ± 0.146	30.911 ± 0.111	30.794 ± 0.579	30.877 ± 0.401
$\chi N = 33$	33.303 ± 0.167	32.893 ± 0.128	32.772 ± 0.649	32.825 ± 0.454
$\chi N = 35$	35.305 ± 0.206	34.833 ± 0.149	34.799 ± 0.831	34.846 ± 0.509
$\chi N = 37$	37.308 ± 0.275	36.779 ± 0.177	36.785 ± 0.826	36.818 ± 0.558
$\chi N = 39$	39.365 ± 0.297	38.898 ± 0.206	38.119 ± 0.612	38.534 ± 0.534

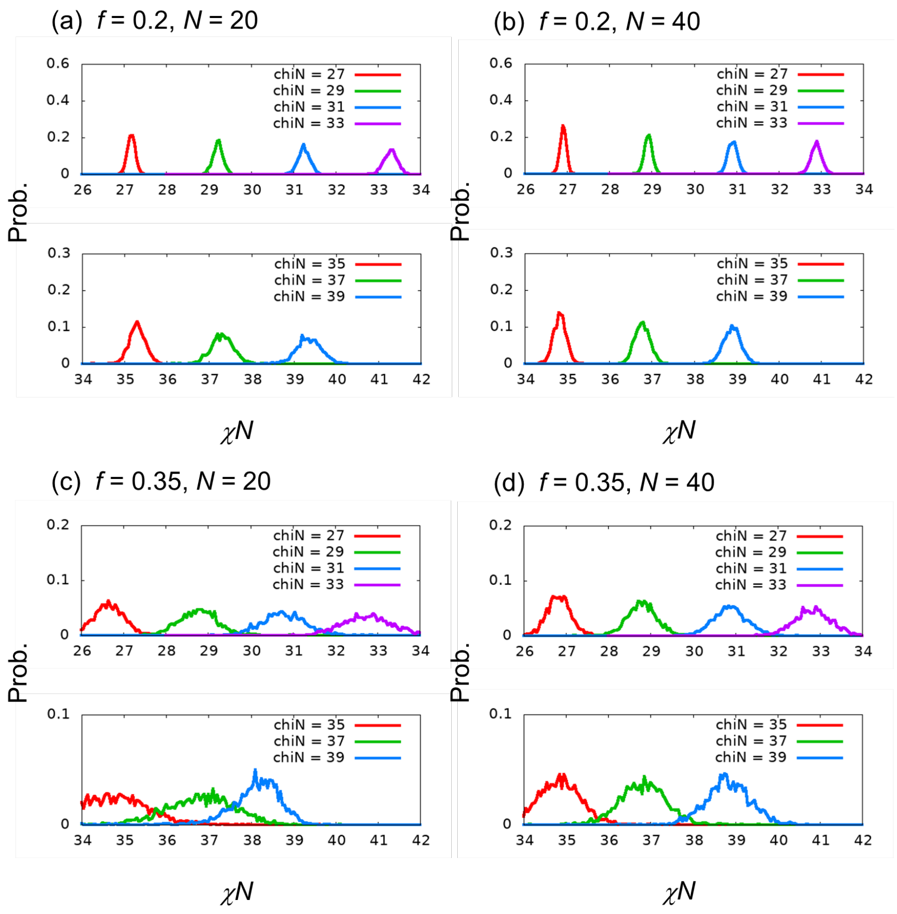

Figure S6. Probability distribution functions of the estimated χN for evaluation data with the unlearned χN values for the training with 50 epochs. Here, the size of each bin was 0.05.

S5. Detailed investigations around $\chi N = 38$ for the 8-class regression problem with 100 epochs

In the 8-class regression problem with 100 epochs for (f, N) = (0.35, 20), we considered that the χN estimation failed for $\chi N > 38$ as presented in Fig. 5 in the main text. To verify this, the behaviors around $\chi N = 38$ were examined. Figure S7 shows the distributions of the estimated χN for the cases with $\chi N = 36.5$, 37.5, 38.5, and 39.5. It was concluded that the estimations for $\chi N = 38.5$ and 39.5 failed in the cases of (f, N) = (0.35, 20).

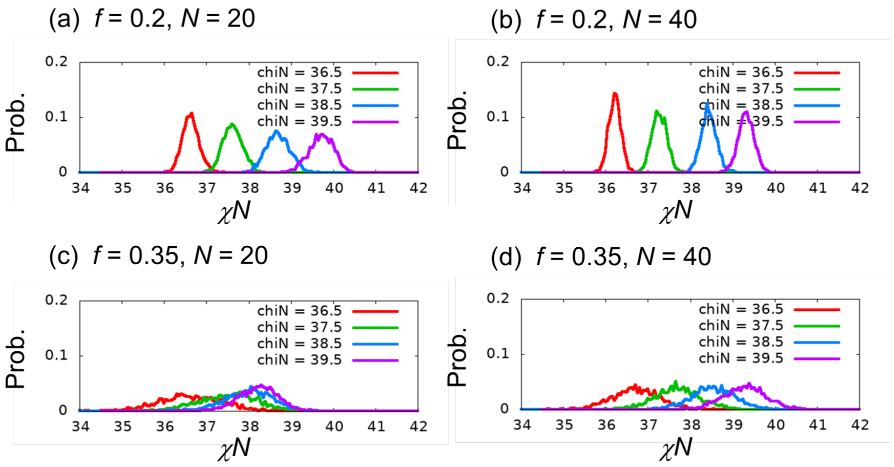

Figure S7. Probability distribution functions of the estimated χN for evaluation data generated with unlearned χN values around $\chi N = 38$. Here, the size of each bin was 0.05.

For (f, N) = (0.35, 20), the results of two independent learnings are presented in Fig. S8. The learning curves were similar among the three independent learning methods. Failures of χN = 39 for (f, N) = (0.35, 20) are commonly observed. Based on this behavior, we believe that the images obtained when $\chi N = 39$ resemble those of $\chi N = 38$.

For verification, we used the image classification problem for three classes of $\chi N = 38$, 39, and 40. Table S12 presents the confusion matrix. We can conclude that it was difficult to distinguish between $\chi N = 38$ and 39.

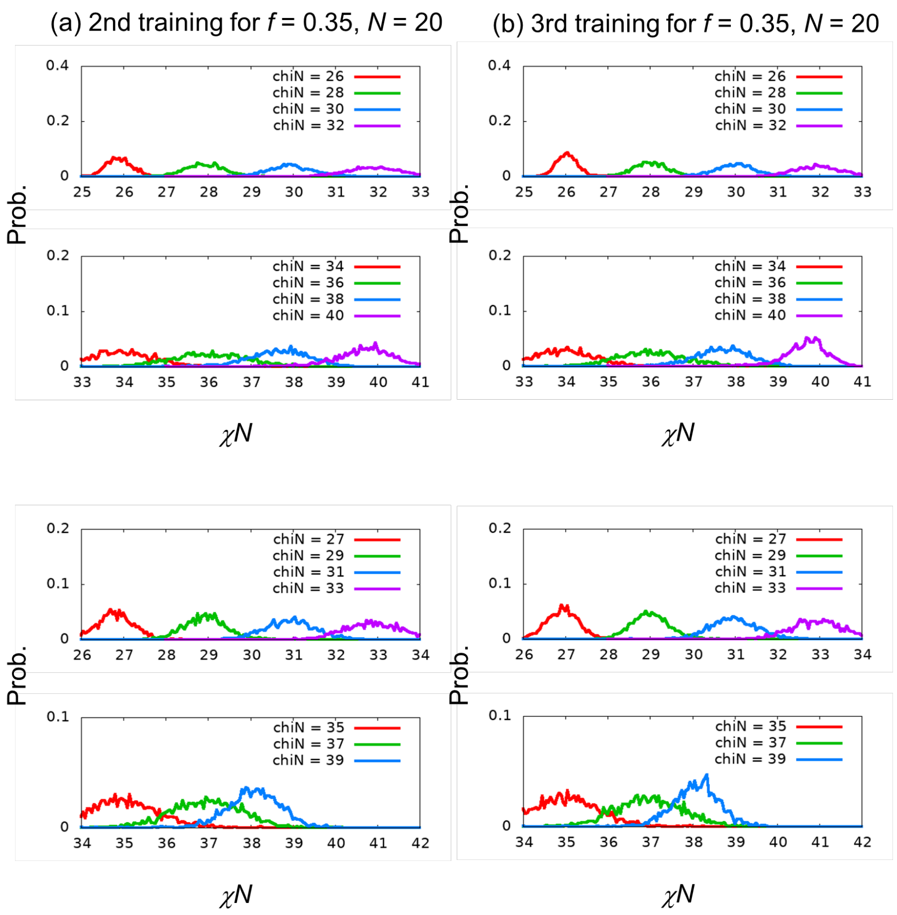

Figure S8. Probability distribution functions of the estimated χN in the (a) second and (b) third trainings. Here, the size of each bin was 0.05.

Table S12. Confusion matrices of the 3-class problem with $\chi N = 38$, 39, and 40 for (f, N) = (0.35, 20).

(a) 1000 epochs		VGG-16 estimated χN class		
		38	39	40
Actual	$\chi N = 38$	1138	860	2
	$\chi N = 39$	971	1029	0
	$\chi N = 40$	12	1	1987
(b) 4000 epochs		VGG-16 estimated χN class		
		30	39	40
Actual	$\chi N = 38$	1155	842	3
	$\chi N = 39$	934	1066	0
	$\chi N = 40$	11	0	1991

S6. Cases with long learning times (epochs)

Training iterations with 1000 epochs were performed to investigate the crossover behavior in overfitting. We also examined cases with 4000 epochs that clearly showed overfitting. The results for the 4000 epochs are presented in the next section S7.

Figure S9 shows the learning curves for 1000 epochs. Due to the discrepancy between the training and evaluation MAEs, overfitting was observed. To investigate the details of the distributions of the estimated χN, they are shown in Figs. S10 and S11.

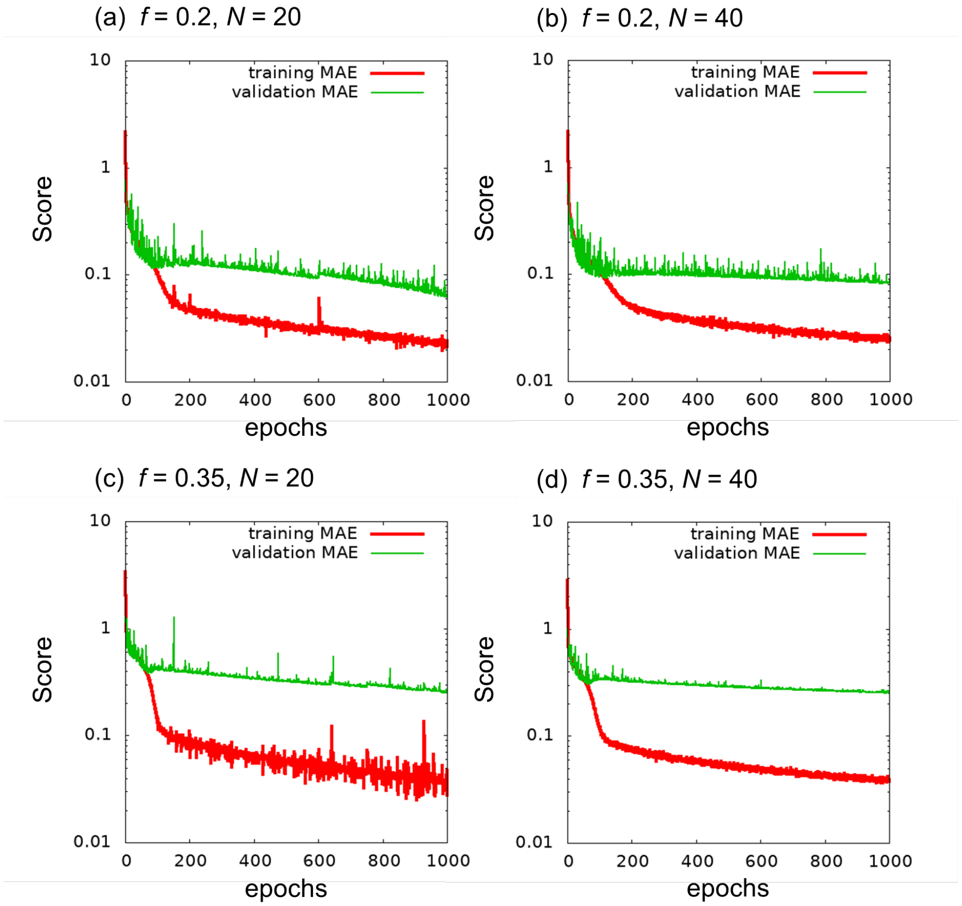

Figure S9. Learning curves of the regression problem until 1000 epochs.

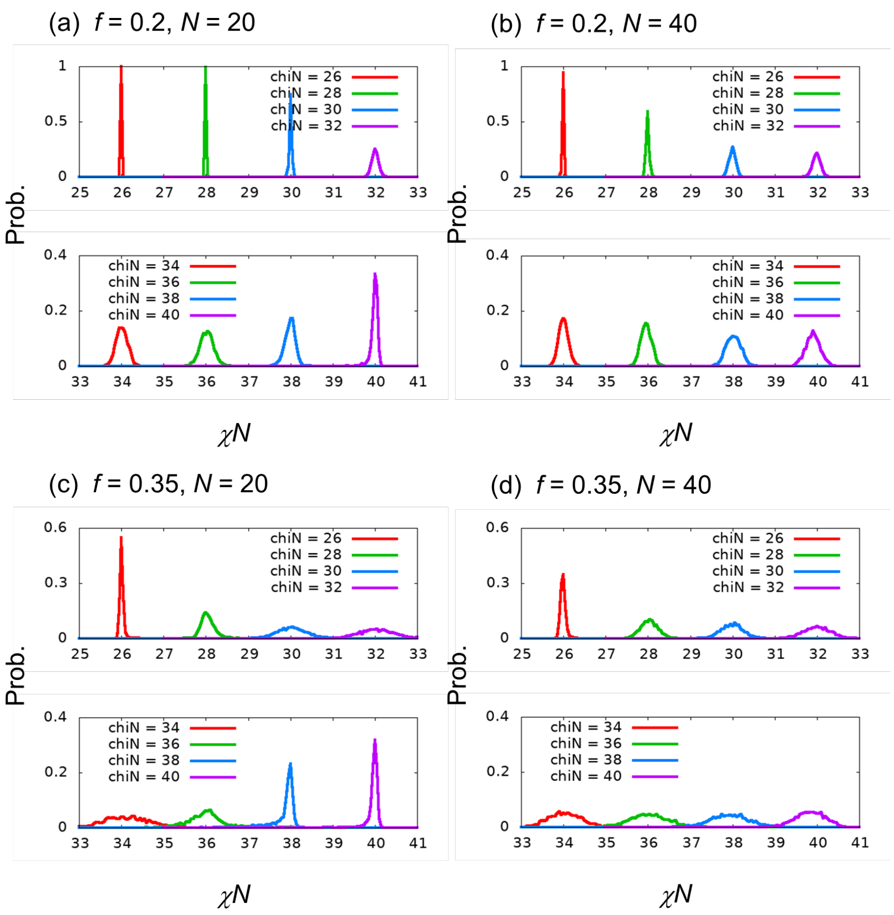

Figure S10. Probability distribution functions of the estimated χN for evaluation data with the same χN values for the training with 1000 epochs. Here, the size of each bin was 0.05.

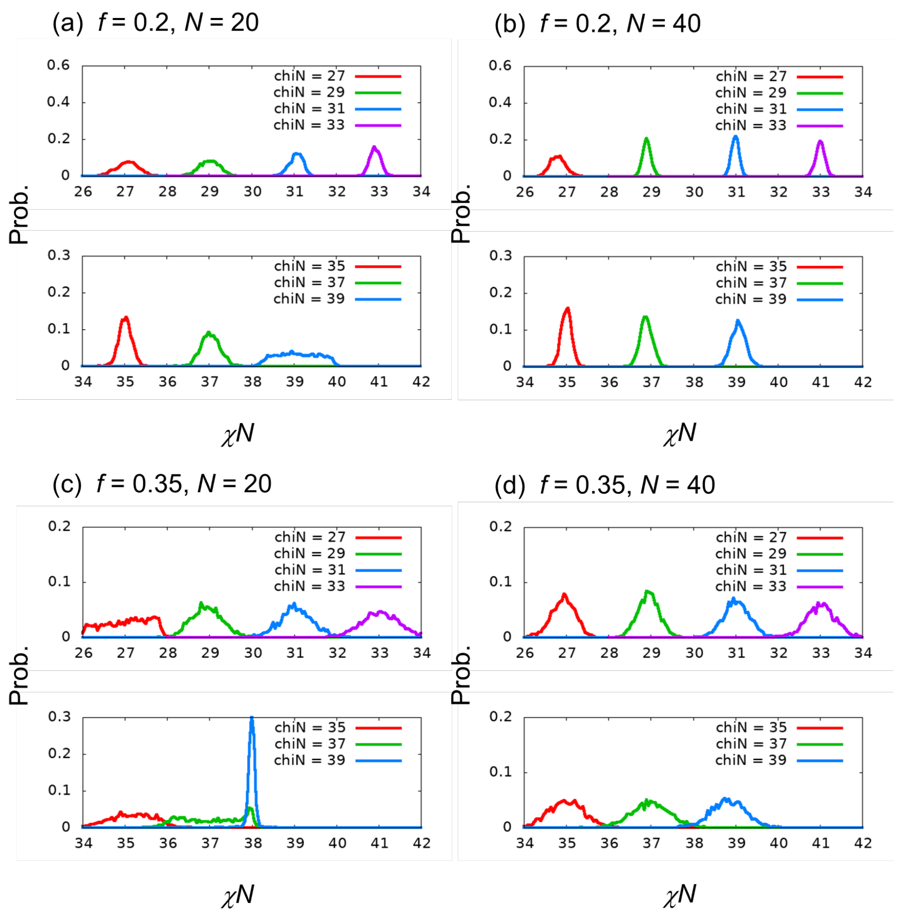

Figure S11. Probability distribution functions of the estimated χN for evaluation data with the unlearned χN for the training with 1000 epochs. Here, the size of each bin was 0.05.

From the obtained behaviors, we found that the tendency of overfitting was observed near both ends of the examined χN range when compared with the central part. Regarding the χN values in overfitting, the distributions of the estimated χN for the same χN class as the teaching images became narrow, and those for the unlearned χN became wide. In particular, we found that the flat distribution was a typical behavior observed in the overfitting states, as shown in Figs. S11 (a) and (c). In addition, as shown in Fig. S11 (c), when $\chi N = 37$, sharp peaks were observed at χN values of the teaching images. For example, in the case of $(\chi N, f, N) = (37, 0.35, 20)$, two peaks at $\chi N = 36$ and 38 are observed in Fig. S11 (c). For the 4000 epochs shown in the next section, sharp peaks were observed.

S7. Behaviors of overfitted networks with much longer learning times

To clarify the behaviors for the overfitting cases, we trained the regression problem until 4000 epochs. Figure S12 shows the learning curve, and Figures S13 and S14 present the distributions of the estimated χN. We observed the typical behavior of failures (sharp peaks at χN values of the teaching images) in the estimations for unlearned χN values.

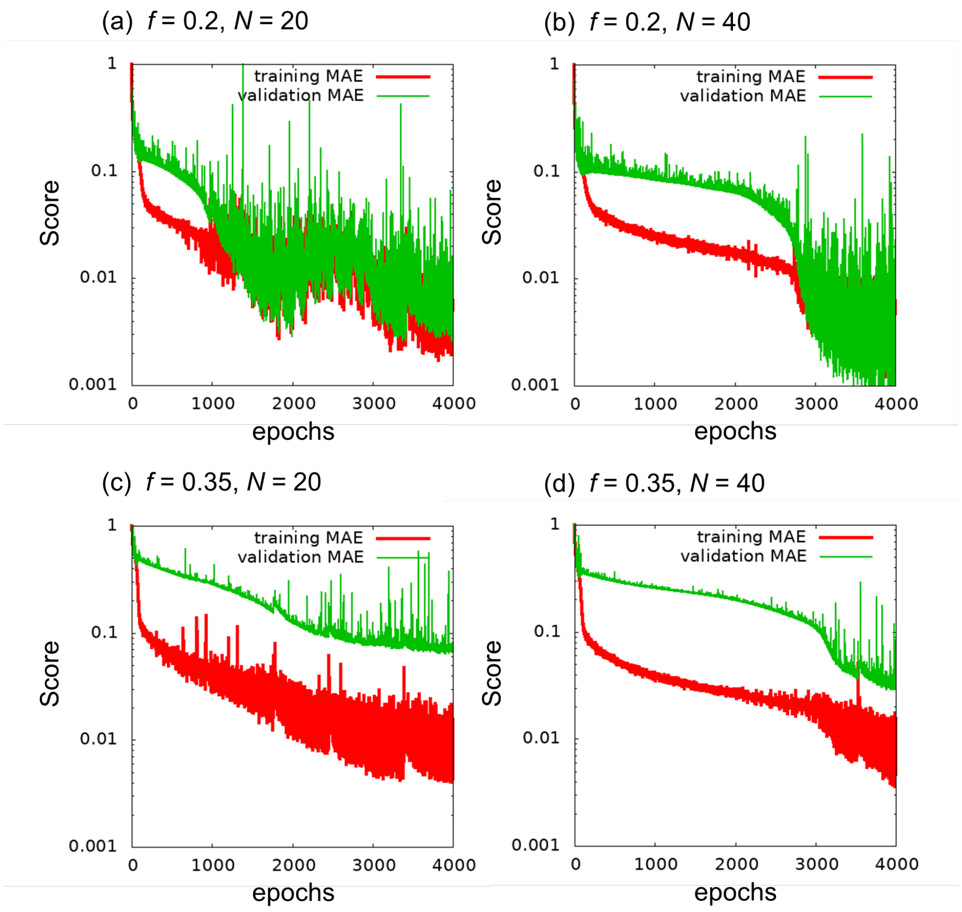

Figure S12. Learning curves of the regression problem until 4000 epochs.

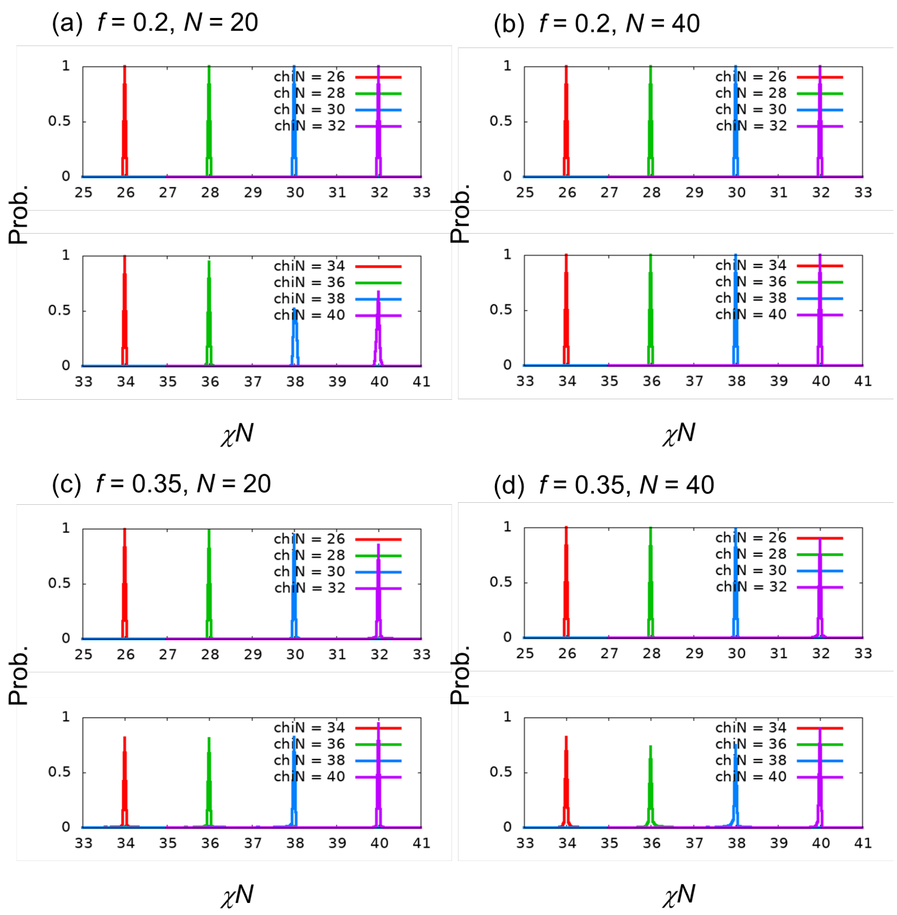

Figure S13. Probability distribution functions of the estimated χN for evaluation data with the same χN values for the training with 4000 epochs. Here, the size of each bin was 0.05.

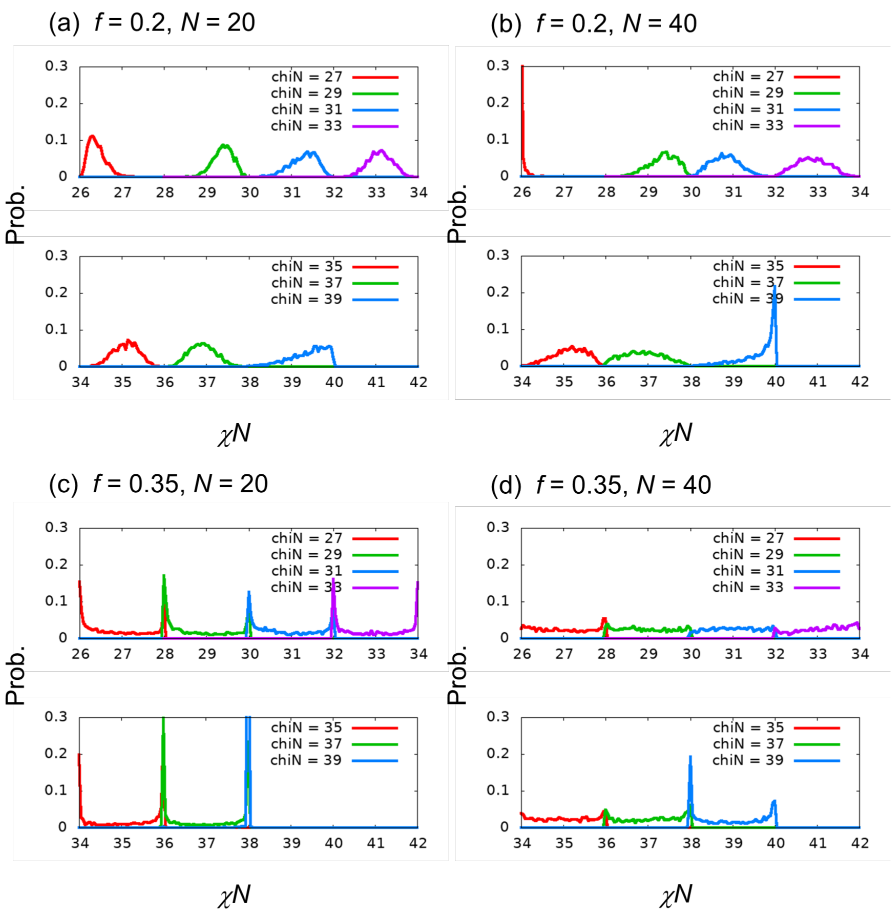

Figure S14. Probability distribution functions of the estimated χN for evaluation data with the unlearned χN values for the training with 4000 epochs. Here, the size of each bin was 0.05.

S8. Transfer learning with frozen network weights of the image classification problem

Transfer learning is a widely used technique for shortening the learning time (epochs) using the already learned network weights as initial weights. For problems in the present work, it is prudent to use the weights learned in the image classification for learning the regression problem. In this section, we describe that the transfer learning is performed by learning only the weights of the full connection layer block, as shown in Fig. 8 (b). Herein, the weight of the convolution layer block of the network shown in Fig. 8 (b) did not change. The results of transfer learning to change all weights, including the convolution layer blocks, are provided in the next

section. The behaviors were similar to those when the weight of the convolution layer blocks was fixed.

Figure S15 shows the learning curves of the transfer learning using the weights learned in the 8-class image classification problem as initial weights. For all cases, the training and evaluation MAEs became smaller when compare to that shown in Fig. 3. We found that transfer learning shortened the learning time. In the case of regression problems, it is also necessary to maintain the generalization performance of the unlearned χN. This can be investigated through the probability distribution functions.

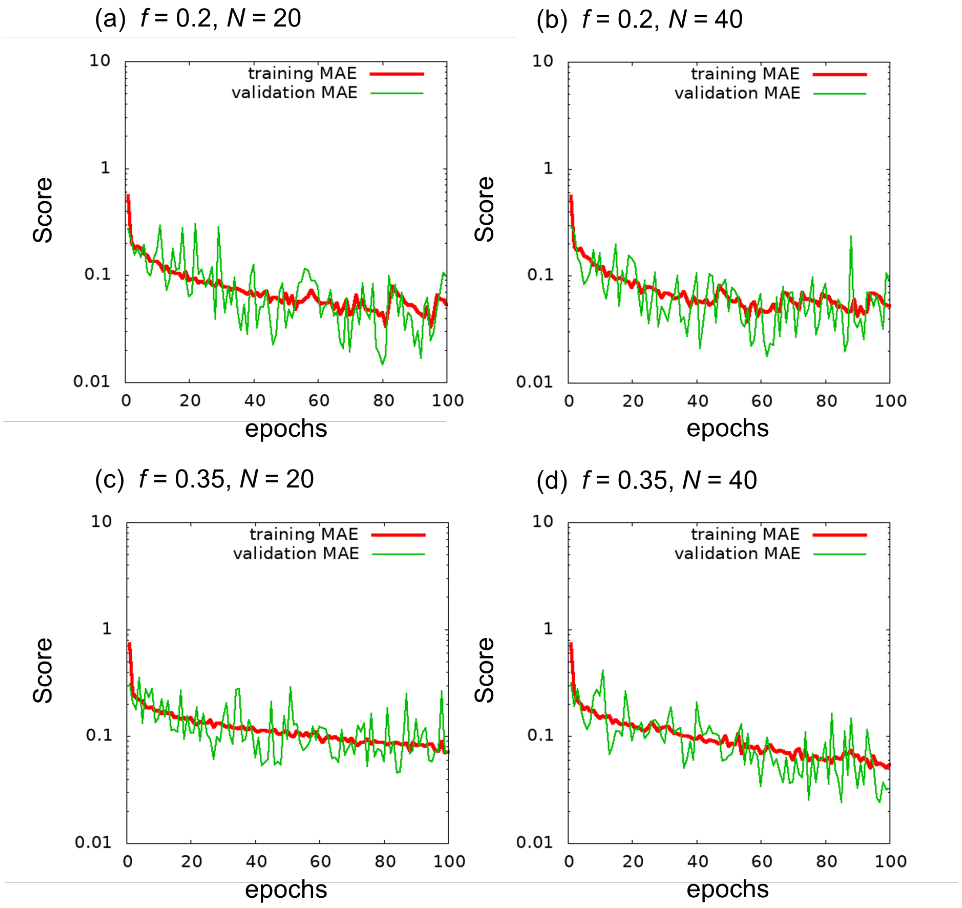

Figure S15. Learning curves of the regression problem until 100 epochs using transfer learning from the classification problem.

Figures S16 and S17 present distributions of the estimated χN for the cases with transfer learning. It was clearly found that the obtained results were typical behaviors (sharp peaks at χN values of the teaching images) of the overfitting.

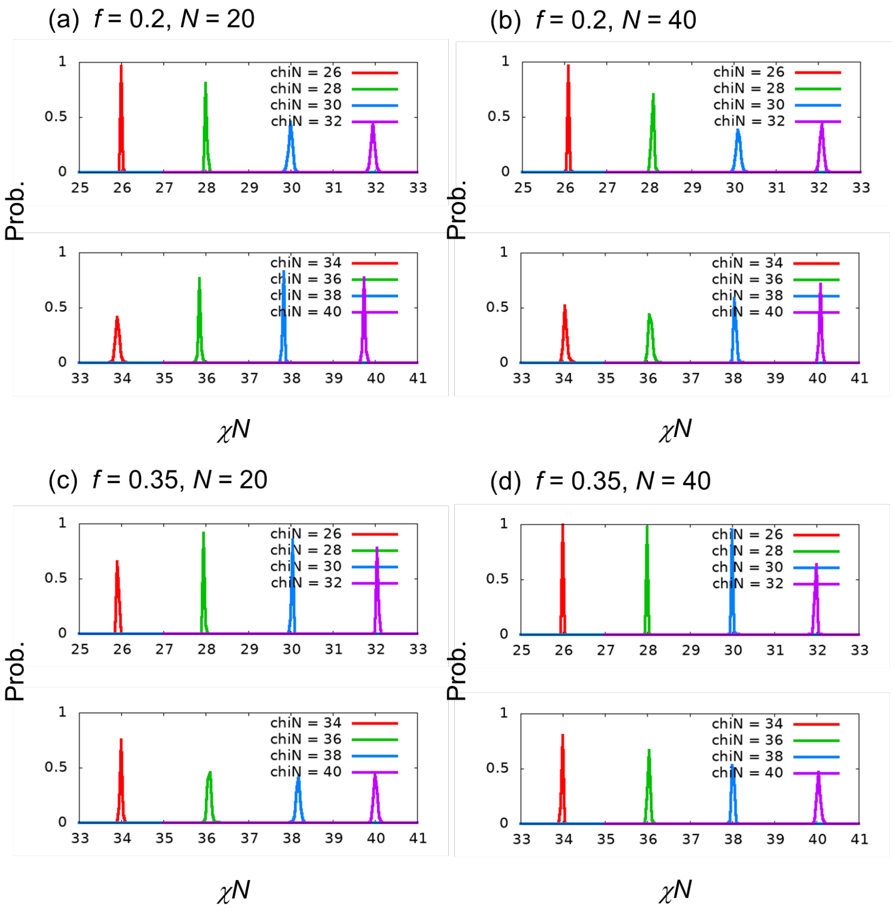

Figure S16. Probability distribution functions of the estimated χN for evaluation data with the same χN values as the training images using transfer learning with 100 epochs from the classification problem. Here, the size of each bin was 0.05.

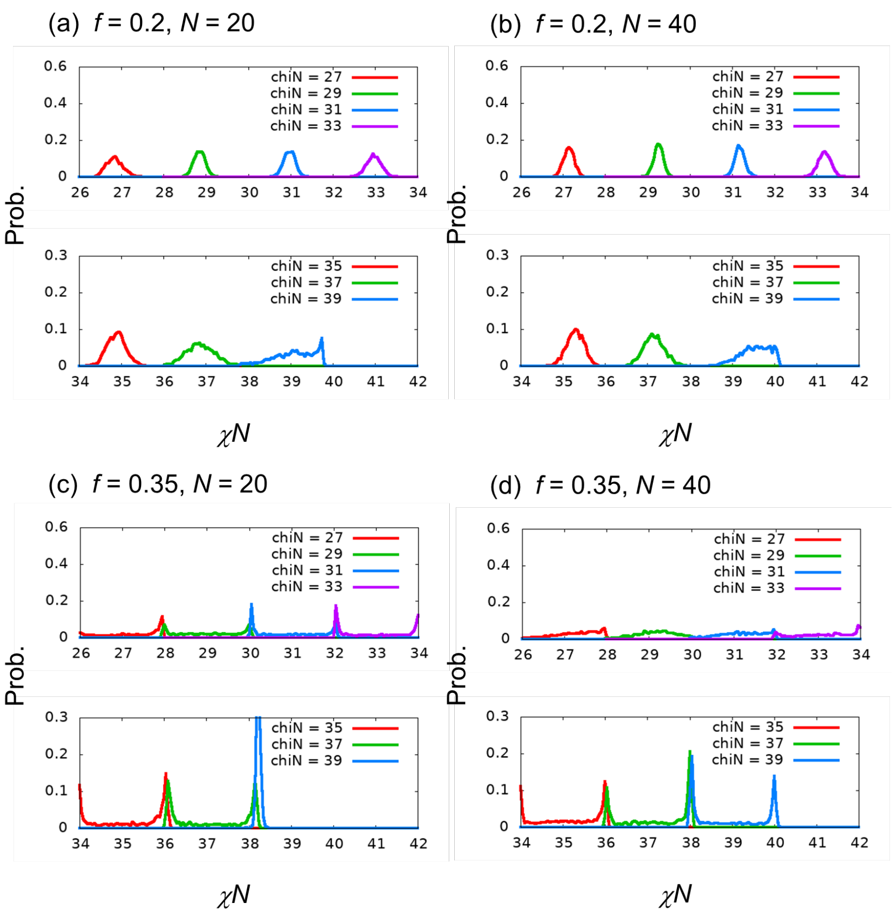

Figure S17. Probability distribution functions of the estimated χN for evaluation data with the unlearned χN using transfer learning with 100 epochs from the classification problem. Here, the size of each bin was 0.05.

S9. Transfer learning using initial weights obtained in the image classification problem

For comparisons of transfer learning without freezing weights, we performed transfer learning for all weights of the network, as shown in Fig. 8 (b). In the main text, the weight of the convolution layer blocks in Fig. 8 (b) was fixed. Figure S18 shows the learning curves and Figures S19 and S20 present the distributions of the estimated χN. Here, the obtained behaviors (sharp peaks at χN values of the teaching images) were similar to those when the weight of the convolution layer block was fixed.

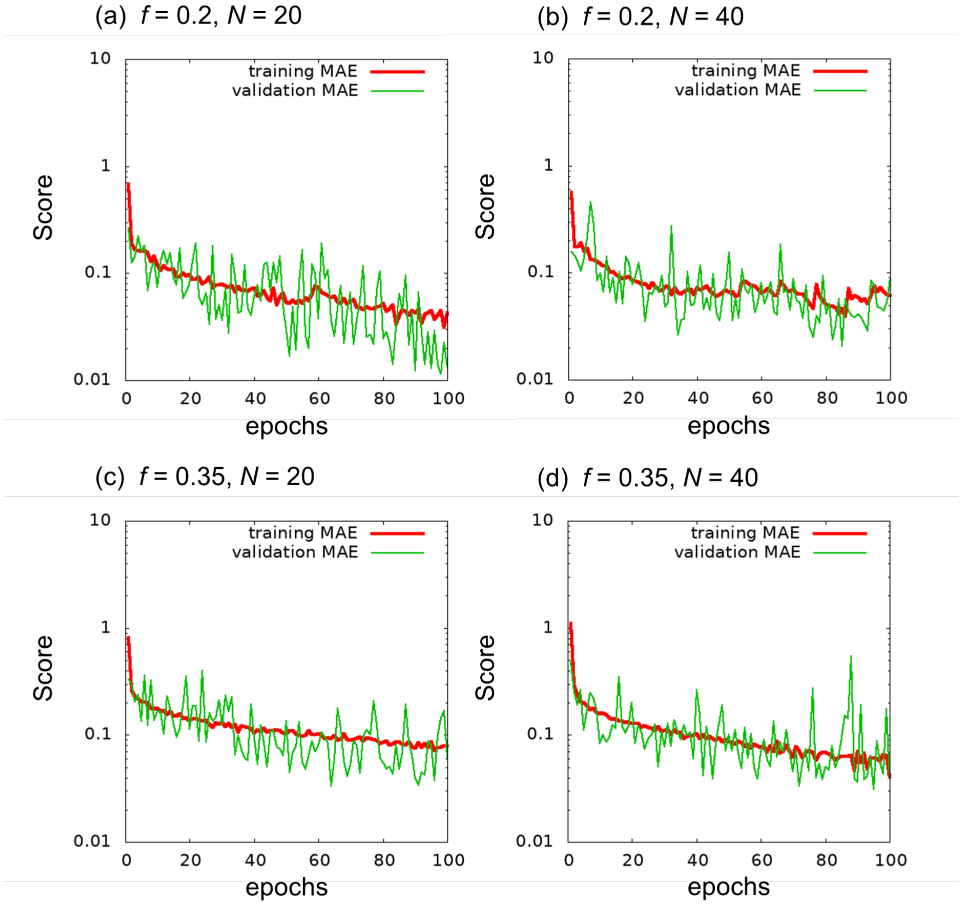

Figure S18. Learning curves of the regression problem until 100 epochs using transfer learning from the classification problem.

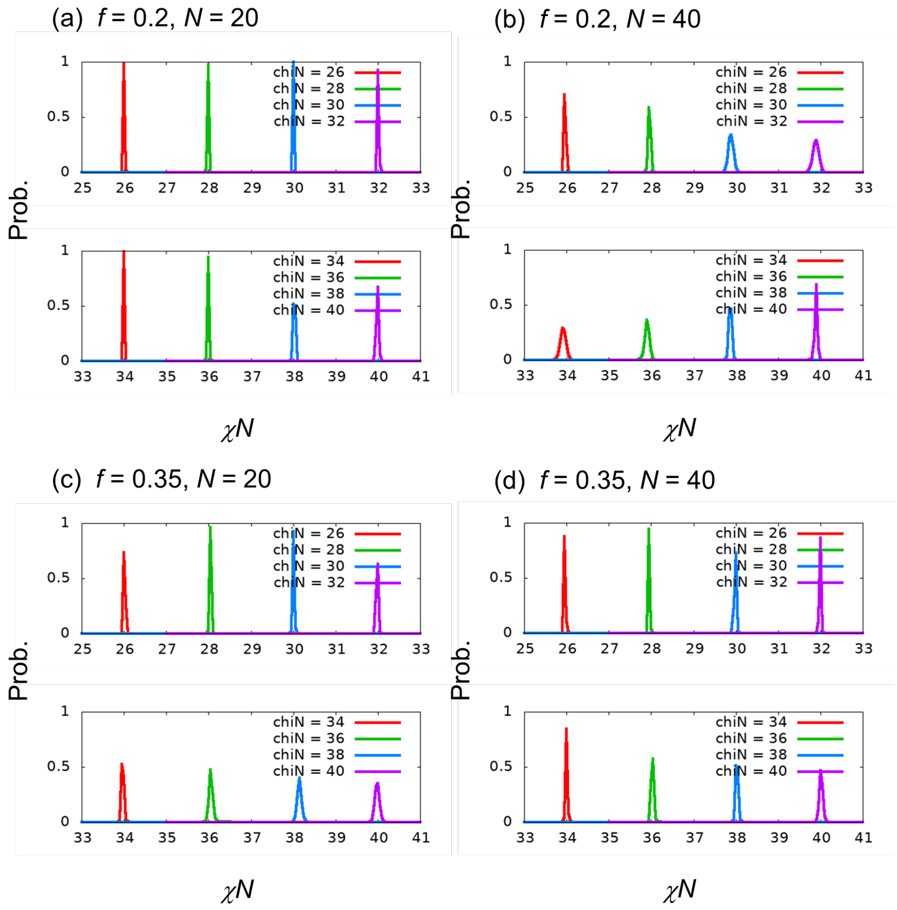

Figure S19. Probability distribution functions of the estimated χN for evaluation data with the same χN values as teaching images using transfer learning with 100 epochs from the classification problem. Here, the size of each bin was 0.05.

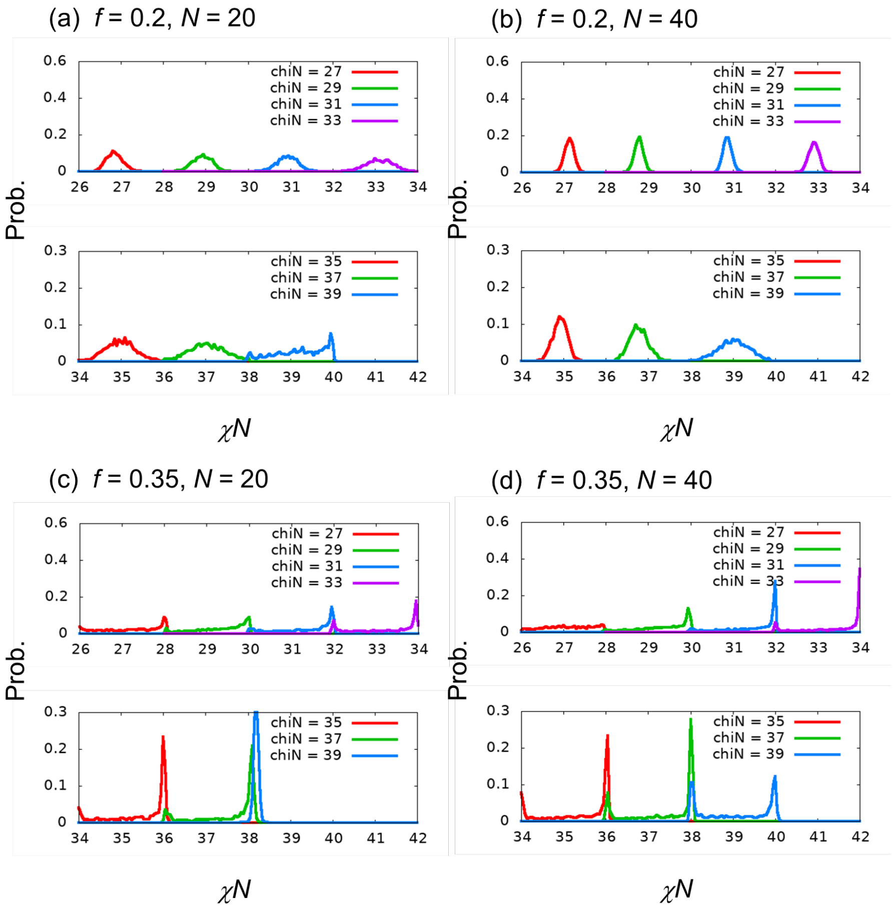

Figure S20. Probability distribution functions of the estimated χN for evaluation data with the unlearned χN values using transfer learning with 100 epochs from the classification problem. Here, the size of each bin was 0.05.

S10. Regression problem for the binarized images

To confirm the effects of the interfacial density gradients on the regression problem, we investigated the performance of regression using binarized images. Figure S21 shows the learning curves for binarized images. The obtained MAEs were found to be worse than those for gray-scale images. Figures S22 and S23 present the distributions of estimated χN. The averages and standard deviations for each χN are listed in Tables 6 and 7 in the main text. The distributions for the binarized images were much wider than those for the gray scale images. Absolute differences from the true values of χN were also larger than those observed for the gray scale images. Therefore, we concluded that the gray scale images provided essential information for estimating χN. This suggests that χN can be evaluated accurately without using DL, if an arithmetic calculation method for estimating χN from a cross-sectional image is developed. At present, the arithmetic calculation method is unknown; therefore, DL is an effective tool.

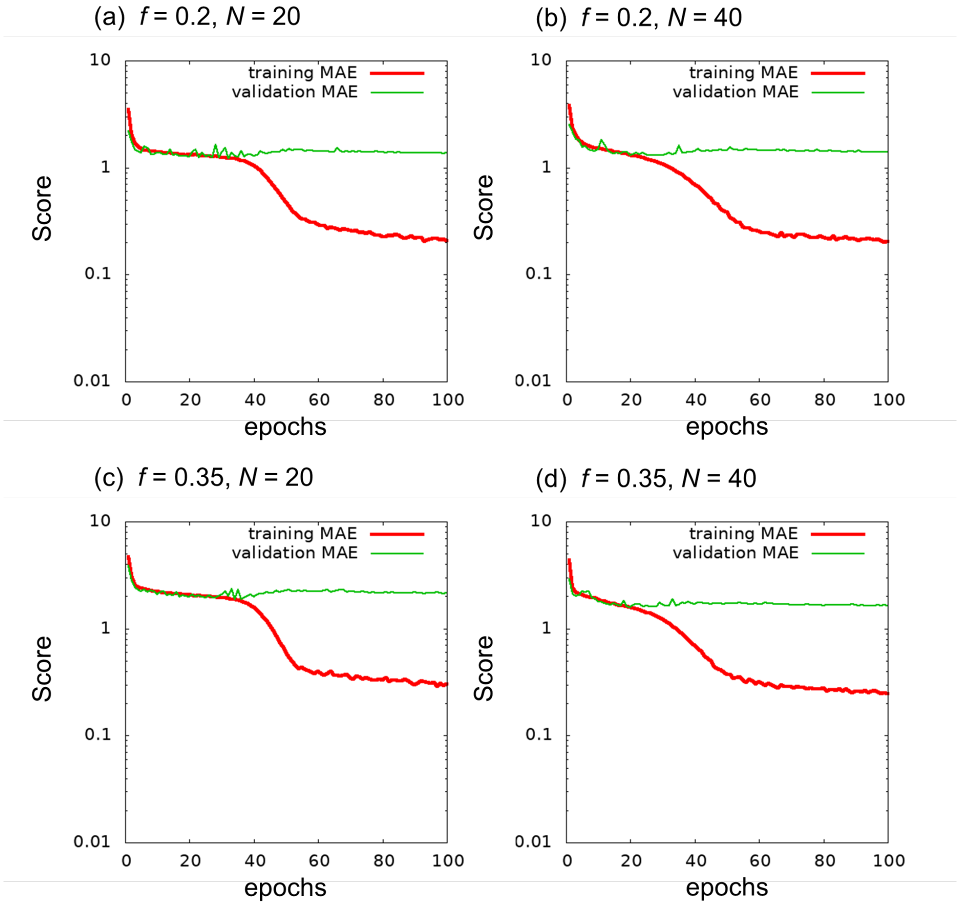

Figure S21. Learning curves of the regression problem for the binarized images.

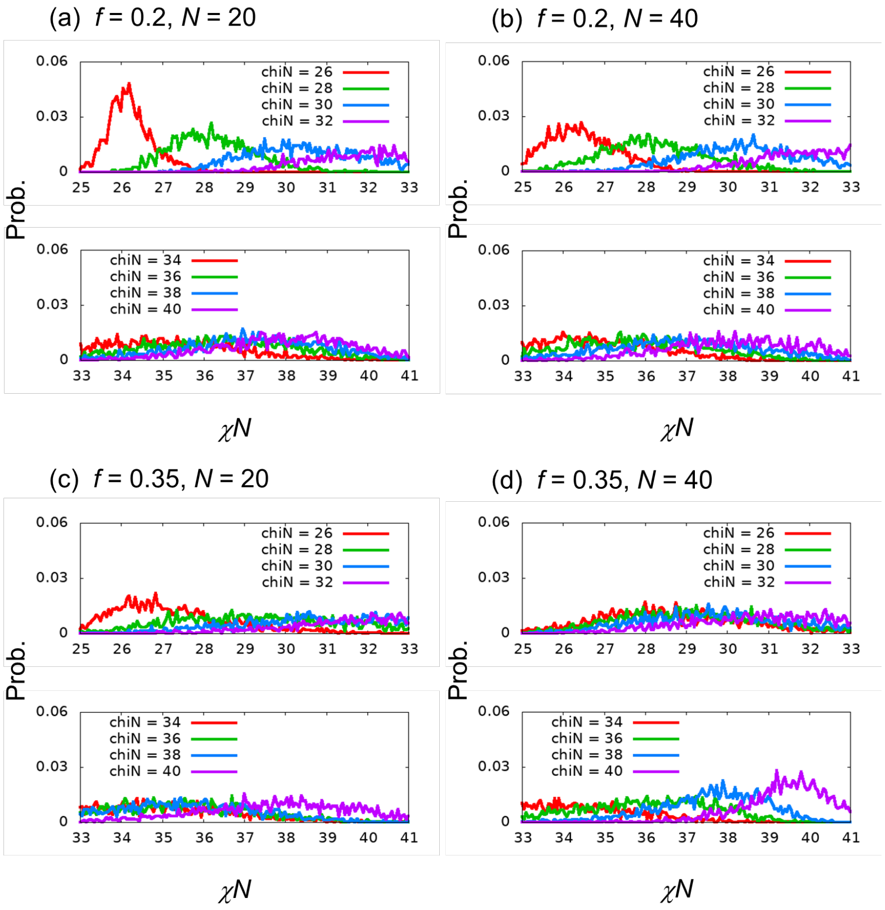

Figure S22. Probability distribution functions of the estimated χN for evaluation data of the binarized images with the same χN values for the training. Here, the size of each bin was 0.05.

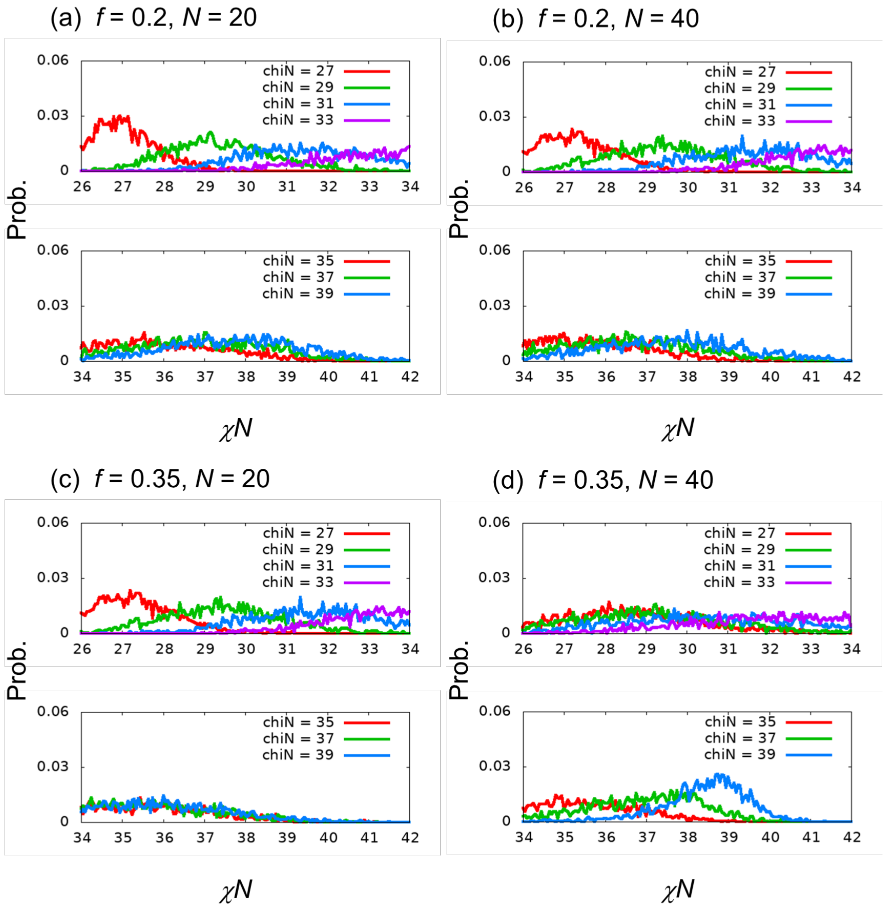

Figure S23. Probability distribution functions of the estimated χN for evaluation data of the binarized images with the unlearned χN values. Here, the size of each bin was 0.05.

S11. SCF calculation

In the real-space SCF theory [52, 53] for an A-B BCP, Gaussian statistics for the chain conformation were assumed owing to the screening effect in the melts. Thus, the K-type (K = A or B) segment is characterized by the effective bond length b_K, and the K-type block is characterized by the number of segments N_K.

The local segment density $\phi_K(\boldsymbol{r})$ of the K-type (K = A or B) segment was obtained by solving a set of self-consistent equations using an iterative refinement method. The self-consistent external potential $V_K(\boldsymbol{r})$ imposed on the K-type segments is decomposed into two contributions: the direct interaction potential imposed by the nearest-neighbor segments and the

constraining potential imposed by the segment density profile $\phi_K(\boldsymbol{r})$. According to the references [52,53], $V_K(\boldsymbol{r})$ is

$$V_K(\boldsymbol{r}) = \sum_{K'} \epsilon_{KK'} \phi_{K'}(\boldsymbol{r}) - \mu_K(\boldsymbol{r})$$

where $\mu_K(\boldsymbol{r})$ is the chemical potential of the K-type segment. The first term represents the interaction energy between segments, where $\epsilon_{KK'}$ is the nearest-neighbor pair interaction energy between a K-type segment and a K'-type segment which is related to the Flory-Huggins interaction parameter via $\chi_{K,K'} = z\beta[\epsilon_{K,K'} - (\epsilon_{K,K} + \epsilon_{K',K'})/2]$, where z is the number of nearest-neighbor sites. $\mu_K(\boldsymbol{r})$ can be regarded as the Lagrange multiplier that fixes the density of the K-type segments at position $\boldsymbol{r}$ to $\phi_K(\boldsymbol{r})$.

The statistical weight of a sub-chain of the K-type from the i-th segment to j-th segment is expressed as

$$Q_K(i, \boldsymbol{r}_i;\ j, \boldsymbol{r}_j) = \frac{1}{z^{|i-j|}} \sum_{all\ conf.} \left[-\beta \left\{ \frac{1}{2} V_K(\boldsymbol{r}_i) + \sum_{k=i+1}^{j-1} V_K(\boldsymbol{r}_k) + \frac{1}{2} V_K(\boldsymbol{r}_j) \right\} \right]$$

where $\boldsymbol{r}_i$ and $\boldsymbol{r}_j$ denote the positions of the i- and j-th segments, respectively. For a small $\beta V_K(\boldsymbol{r}_j)$, the statistical weight was governed by following the Edwards equation:

$$\frac{\partial}{\partial i} Q_K(i, \boldsymbol{r}_i;\ j, \boldsymbol{r}_j) = \left[\frac{b^2}{6} \nabla^2 - \beta V_K(\boldsymbol{r}_j) \right] Q_K(i, \boldsymbol{r}_i;\ j, \boldsymbol{r}_j)$$

where b is the Kuhn length. The density field of the K-type segment at $\boldsymbol{r}$ was obtained using the following expression

$$\phi_K(\boldsymbol{r}) = C_K \sum_i \int d\boldsymbol{r}_0 \int d\boldsymbol{r}_{N_K}\, Q_K(0, \boldsymbol{r}_0;\ j, \boldsymbol{r}_j) Q_K(i, \boldsymbol{r}_i;\ N_K, \boldsymbol{r}_{jN_K})$$

where C_K denotes the normalized coefficient and N_K is the total number of segments in a chain.

$V_K(\boldsymbol{r})$, $Q_K(i, \boldsymbol{r}_i;\ j, \boldsymbol{r}_j)$ and $\phi_K(\boldsymbol{r})$ must be determined in a self-consistent manner by iterative refinements of these variables. The solver of the SCF calculation was provided by the OCTA/SUSHI package [92, 93].

S12. Comparisons among popular network models

As a preliminary study, we compared the generalization abilities of VGG-16 [39], VGG-19 [39], ResNet-50 [40], Xception [42], MobileNet [43], and DenseNet-121 [44]. We evaluated the confusion matrix $M_{i,j}$ and error rates E after learning with 100 epochs for 4-class image-classification problems using f = 0.2 and 0.35 and with χN = 25, 30, 35, and 40. Herein, for the confusion matrix $M_{i,j}$, accuracy A and error rate was calculated using the expressions, $A = \sum_i M_{i,i} / \sum_{i,j} M_{i,j}$, and $E = 1 - A$, respectively.

Table S13 lists the error rates E for (f, N) = (0.2, 20), (0.2, 40), (0.35, 20), and (0.35, 40) obtained using VGG-16, VGG-19, ResNet-50, Xception, MobileNet, and DenseNet-121 network models. From the results, it was found that VGG-16 showed the best performance. The confusion matrices with the above-mentioned six network models are listed in Tables S14–S17. From the obtained results, all network models were showed good results. VGG-16, VGG-19, and DenseNet-121 showed better performance than ResNet-50, Xception, and MobileNet. Because VGG-16 is the simplest network model, we used it in the main text. Notably, the performance comparison among the models depended on the amount of data, learning time, and several hyper parameters.

Table S13. Error rates for (f, N) = (0.2, 20), (0.2, 40), (0.35, 20), and (0.35, 40).

	(f, N) = (0.2, 20)	(f, N) = (0.2, 40)	(f, N) = (0.35, 20)	(f, N) = (0.35, 40)
VGG-16	1.25×10^{-4}	0.00	1.13×10^{-2}	1.63×10^{-3}
VGG-19	1.25×10^{-4}	0.00	2.68×10^{-2}	1.50×10^{-3}
ResNet-50	8.00×10^{-3}	9.63×10^{-3}	5.03×10^{-2}	9.38×10^{-3}
Xception	1.38×10^{-3}	5.00×10^{-4}	3.64×10^{-2}	7.00×10^{-3}
MobileNet	1.25×10^{-3}	2.50×10^{-4}	1.37×10^{-1}	7.00×10^{-3}
DenseNet-121	7.25×10^{-3}	0.00	8.88×10^{-3}	1.78×10^{-2}

Table S14. Confusion matrix for (f, N) = (0.2, 20).

		VGG-16 estimated χN class				VGG-19 estimated χN class			
		25	30	35	40	25	30	35	40
Actual	$\chi N = 25$	2000	0	0	0	2000	0	0	0
	$\chi N = 30$	0	2000	0	0	0	2000	0	0
	$\chi N = 35$	0	0	1999	1	0	0	1999	1
	$\chi N = 40$	0	0	0	2000	0	0	0	2000
		ResNet-50 estimated χN class				Xception estimated χN class			
		25	30	35	40	25	30	35	40
Actual	$\chi N = 25$	2000	0	0	0	2000	0	0	0
	$\chi N = 30$	0	1999	1	0	0	1999	1	0
	$\chi N = 35$	0	6	1989	5	0	0	1997	3
	$\chi N = 40$	0	0	52	1948	0	0	7	1993
		MobileNet estimated χN class				DenseNet-121 estimated χN class			
		25	30	35	40	25	30	35	40
Actual	$\chi N = 25$	2000	0	0	0	2000	0	0	0
	$\chi N = 30$	0	2000	0	0	0	2000	0	0
	$\chi N = 35$	0	0	1999	1	0	0	2000	0
	$\chi N = 40$	0	0	9	1991	0	0	58	1942

Table S15. Confusion matrix for (f, N) = (0.2, 40).

		VGG-16 estimated χN class				VGG-19 estimated χN class			
		25	30	35	40	25	30	35	40
Actual	$\chi N = 25$	2000	0	0	0	2000	0	0	0
	$\chi N = 30$	0	2000	0	0	0	2000	0	0
	$\chi N = 35$	0	0	2000	0	0	0	2000	0
	$\chi N = 40$	0	0	0	2000	0	0	0	2000
		ResNet-50 estimated χN class				Xception estimated χN class			
		25	30	35	40	25	30	35	40
Actual	$\chi N = 25$	2000	0	0	0	2000	0	0	0
	$\chi N = 30$	0	2000	0	0	0	2000	0	0
	$\chi N = 35$	0	9	1991	0	0	0	2000	0
	$\chi N = 40$	0	0	68	1932	0	0	4	1996
		MobileNet estimated χN class				DenseNet-121 estimated χN class			
		25	30	35	40	25	30	35	40
Actual	$\chi N = 25$	2000	0	0	0	2000	0	0	0
	$\chi N = 30$	0	2000	0	0	0	2000	0	0
	$\chi N = 35$	0	0	2000	0	0	0	2000	0
	$\chi N = 40$	0	0	2	1998	0	0	0	2000

Table S16. Confusion matrix for (f, N) = (0.35, 20).

		VGG-16 estimated χN class				VGG-19 estimated χN class			
		25	30	35	40	25	30	35	40
Actual	$\chi N = 25$	1985	15	0	0	1995	5	0	0
	$\chi N = 30$	0	1967	33	0	0	1928	72	0
	$\chi N = 35$	1	2	1966	31	1	3	1869	127
	$\chi N = 40$	0	0	8	1992	0	0	6	1994
		ResNet-50 estimated χN class				Xception estimated χN class			
		25	30	35	40	25	30	35	40
Actual	$\chi N = 25$	1969	31	0	0	2000	0	0	0
	$\chi N = 30$	3	1799	198	0	2	1992	6	0
	$\chi N = 35$	0	43	1868	89	1	87	1906	6
	$\chi N = 40$	0	0	38	1962	0	1	188	1811
		MobileNet estimated χN class				DenseNet-121 estimated χN class			
		25	30	35	40	25	30	35	40
Actual	$\chi N = 25$	1999	1	0	0	2000	0	0	0
	$\chi N = 30$	10	1988	2	0	0	1994	6	0
	$\chi N = 35$	1	734	1260	0	0	54	1935	11
	$\chi N = 40$	0	1	339	1660	0	0	0	2000

Table S17. Confusion matrix for (f, N) = (0.35, 40).

		VGG-16 estimated χN class				VGG-19 estimated χN class			
		25	30	35	40	25	30	35	40
Actual	$\chi N = 25$	1999	1	0	0	1999	1	0	0
	$\chi N = 30$	2	1997	1	0	0	2000	0	0
	$\chi N = 35$	0	2	1992	6	0	1	1991	8
	$\chi N = 40$	0	0	1	1999	0	0	2	1998
		ResNet-50 estimated χN class				Xception estimated χN class			
		25	30	35	40	25	30	35	40
Actual	$\chi N = 25$	2000	0	0	0	2000	0	0	0
	$\chi N = 30$	6	1990	4	0	1	1999	0	0
	$\chi N = 35$	0	40	1953	7	0	11	1988	1
	$\chi N = 40$	0	0	108	1982	0	0	43	1957
		MobileNet estimated χN class				DenseNet-121 estimated χN class			
		25	30	35	40	25	30	35	40
Actual	$\chi N = 25$	1999	1	0	0	2000	0	0	0
	$\chi N = 30$	0	1998	2	0	0	2000	0	0
	$\chi N = 35$	0	4	1985	11	0	58	1942	0
	$\chi N = 40$	0	0	38	1962	0	0	84	1916
